# Patterns of antimicrobial resistance in *Streptococcus suis* isolates from pigs with or without streptococcal disease in England between 2009 and 2014

**DOI:** 10.1016/j.vetmic.2017.06.002

**Published:** 2017-08

**Authors:** Juan Hernandez-Garcia, Jinhong Wang, Olivier Restif, Mark A. Holmes, Alison E. Mather, Lucy A. Weinert, Thomas M. Wileman, Jill R. Thomson, Paul R. Langford, Brendan W. Wren, Andrew Rycroft, Duncan J. Maskell, Alexander W. Tucker, on behalf of the BRADP1T Consortium

**Affiliations:** a Department of Veterinary Medicine, University of Cambridge, Madingley Road, Cambridge, CB3 0ES, UK; b SAC Consulting: Veterinary Services, Bush State, Penicuik, EH26 0QE, Scotland, UK; c Section of Paediatrics, Department of Medicine, Imperial College London, St. Mary's Campus, London, W2 1 PG, UK; d Faculty of Infectious & Tropical Diseases, London School of Hygiene & Tropical Medicine, Keppel Street, London, WC1E 7HT, UK; e The Royal Veterinary College, Hawkshead Campus, Hatfield, Hertfordshire, AL9 7TA, UK

**Keywords:** Antimicrobial resistance, *Streptococcus suis*

## Abstract

Antimicrobial resistance in *Streptococcus suis*, a global zoonotic pathogen of pigs, has been mostly studied only in diseased animals using surveys that have not evaluated changes over time.

We compared patterns of resistance between *S. suis* isolates from clinical cases of disease (CC) and non-clinical case (NCC) pigs in England, collected over two discrete periods, 2009–2011 and 2013–2014. Minimum inhibitory concentrations (MIC) of 17 antimicrobials (nine classes) were determined on 405 *S. suis* isolates categorised by sampling period and disease association to assess changes in resistance over time and association with disease. First, isolates were characterized as resistant or susceptible using published clinical breakpoints. Second, epidemiological cut-offs (ECOFF) were derived from MIC values, and isolates classified as wild type (WT) below the ECOFF and non-wild type (NWT) above the ECOFF. Finally, isolate subsets were analysed for shifts in MIC distribution.

NCC isolates were more resistant than CC isolates to cephalosporins, penams, pleuromutilins, potentiated sulphonamides and tetracyclines in both study periods. Resistance levels among CC isolates increased in 2013–2014 relative to 2009–2011 for antimicrobials including aminoglycosides, cephalosporins, fluoroquinolones, pleuromutilins, potentiated sulphonamides and tetracyclines. The prevalence of isolates categorised as NWT for five or more classes of antimicrobials was greater among NCC than CC isolates for both time periods, and increased with time. This study used standardised methods to identify significant shifts in antimicrobial resistance phenotypes of *S. suis* isolated from pigs in England, not only over time but also between isolates from known clinical cases or disease-free pigs.

## Highlights

• Disease and non-disease associated isolates had different resistance profiles.
• Antimicrobial resistance increased over time among clinical isolates.
• Prevalence of isolates with resistance to multiple antimicrobials is increasing.
• Combination of different approaches enhances the information obtained from the data.

## 1 Introduction

*Streptococcus suis* (*S. suis*) is a global pig pathogen which has a major impact on productivity, antimicrobial use and pig welfare (Gottschalk, 2012). Human disease due to *S. suis* was first described in Europe in the 1950s (Wertheim et al., 2009). In Great Britain, *S. suis* is one of the most common causes of systemic disease in post-weaned pigs to be reported by diagnostic laboratories in recent years, resulting in septicemia, meningitis, pneumonia and arthritis.

There is marked and large diversity among *S. suis* strains, with 33 serotypes based on capsular polysaccharides (Gottschalk, 2012), and many non-serotypable strains exist, but most clinical cases are caused by a small number of serotypes. Disease associated strains are characterized by an ensembles of a diverse group of virulence related genes, which may vary geographically, and other genomic features but other strains with apparently low pathogenic potential can be isolated widely as part of the microbiota in the respiratory tract and tonsils of pigs without streptococcal disease (Weinert et al., 2015).

Over the past decade, an increasing level of antimicrobial resistance has been noted in food-borne and other pathogens (Palmieri et al., 2011). This has been recognized as a global problem for public health and the worldwide emergence of multidrug-resistant phenotypes is causing increasing concern (O'Neill, 2016). Antimicrobial resistance profiles, and genetic determinants regulating resistance mechanisms, have been studied in isolates of *S. suis* from pigs and, to a lesser extent, from human cases (Palmieri et al., 2011). Penicillin resistance in *S. suis* was first reported in the UK from a serotype 2 isolate from a human in 1980 (Shneerson et al., 1980) and has emerged in *S. suis* isolates from pigs worldwide (Zhang et al., 2008, Callens et al., 2013). More recently, resistance to third-generation cephalosporins was reported in China and Europe (Hu et al., 2011, Zhang et al., 2015; van Hout et al., 2016). Extensive resistance has been reported against aminoglycosides (Holden et al., 2009, Hu et al., 2011, Palmieri et al., 2011), β-lactams, trimethoprim and amphenicols (Wisselink et al., 2006, Holden et al., 2009, Hu et al., 2011, Ge et al., 2012).

Resistance mechanisms in *S. suis* include new gene acquisition and gene expression modifications, as described for tetracyclines, macrolides, lincomycin, streptogramin B (Palmieri et al., 2011, Chen et al., 2013) and fluoroquinolones (Escudero et al., 2011). Other mechanisms based on gene mutations have been described for tiamulin, quinolones and penicillin (Martel et al., 2001, Gurung et al., 2015). However, other reasons underlying ineffective responses to antimicrobial treatment of *S. suis* disease might include biofilm formation and the production of persistent cells (Seitz et al., 2016). Although reports from different parts of the world indicate widespread clinical resistance in *S. suis* to diverse antimicrobials (Aarestrup et al., 1998, Callens et al., 2013, Varela et al., 2013, de Jong et al., 2014, Zhang et al., 2015; van Hout et al., 2016), there have been no systematic comparisons of antimicrobial susceptibility for *S. suis* isolates collected from pig populations in the same geographic area at different time points using standardised methodology.

Current efforts to improve provision of surveillance data to allow monitoring and international comparisons of antimicrobial resistance for *S. suis* are hampered by differences in testing methodologies and interpretation criteria that are subjective. Standardized methods and cut-offs have been proposed by the Clinical and Laboratorial Standards Institute (CLSI) and the European Committee on Antimicrobial Susceptibility Testing (EUCAST), but the need remains for better harmonization and normalization of results (Kronvall, 2010, Kahlmeter, 2015). Furthermore, clinical breakpoints are not defined for most of the antimicrobials; the literature reports of antimicrobial resistance in *S. suis* apply different clinical breakpoints, which further complicates comparisons of results from different studies. Given these limitations, antimicrobial resistance phenotypes for bacteria have also been studied by determining minimum inhibitory concentration (MIC) values and by categorizing isolates according to epidemiological cut-off (ECOFF) values for each antimicrobial.

This study describes the comparative phenotypic antimicrobial resistance characteristics of 405 isolates of *S. suis* from commercial slaughter pigs in England, representing carefully catalogued isolates of known disease-associated or non-disease associated provenance, from two time periods (2009–2011 and 2013–2014).

## 2 Materials and methods

### 2.1 Sample collection

A total of 405 isolates of *S. suis* were obtained covering two periods 2009–2011 and 2013–2014. These were further split into two classes: disease associated clinical cases (CC) and non-disease associated non-clinical cases (NCC). Disease-associated CC isolates from both 2009–2011 (N = 93, from 83 different laboratory submissions) and 2013–2014 (N = 117, from 113 different laboratory submissions) were cultured from lung, meninges, or other systemic sites of pigs between weaning and slaughter age (1–5 months) with clinical signs and/or gross pathology consistent with *S. suis* infection (including meningitis, septicaemia, arthritis, pneumonia) submitted from pig farms from different geographic locations in England to Animal and Plant Health Agency (APHA) veterinary investigation centres (VICs).

Non-disease associated NCC isolates from 2009 to 2011 (N = 66 from 44 different laboratory submissions) were cultured from tonsils or tracheobronchial swabs of pigs between weaning and slaughter age from different geographic locations in England submitted to the APHA VICS for post-mortem examination in which *S. suis* disease was not diagnosed. None of the clinical histories of these cases reported streptococcal disease at the time of submission. NCC isolates from 2013 to 2014 (N = 129 from 113 pigs) originated from nine breeding sources in the East of England which reported no *S. suis* related clinical signs at the time; these isolates were obtained from 250 tonsils scrapes, 125 from 5 week old pigs and 125 from 20 week old pigs, and submitted to the Scottish Agricultural College (SAC) veterinary laboratories for isolation of *S. suis.* Antimicrobial treatments prior to sample collection were not considered in this study.

The NCC isolates from 2009 to 2011 were isolated by inoculating the samples from pigs onto Columbia agar containing 5% (v/v) sheep blood (TCS biosciences Ltd., Bucks, UK) and incubating at 37 °C in aerobic conditions for up to 48 h. Up to three suspect *S. suis* colonies were selected from each plate based on α-haemolysis and colony morphology, then sub-cultured and tested in pure culture with a biochemical profiling kit (API 32-Strep, Bio-Mérieux, Mercy-l’Étoile, France).

For the NCC samples collected in 2013–2014, three colonies were selected per inoculated plate; API biochemical profile was done and *S. suis* colonies from the same plate presenting the same biochemical profile were considered the same strain so just one of them was selected for the final collection and stored at −80 °C until testing. NCC isolates collected in 2013–2014 were epidemiologically related as they came from the same production pyramid, some of them came from the same farm, and some tonsillar scrapes yielded more than one isolate, which reduces this collection representativeness. In contrast, most of the NCC samples collected in 2009–2011, and the CC samples in both periods, represented cases submitted from pig producers located in different geographic areas in England.

### 2.2 Antimicrobial susceptibility testing

MIC were determined using the micro-broth dilution method, at Quotient Bioresearch, Fordham, UK in accordance the CLSI Approved standard M100-S25 (2015), VET01-A4 (2013b) and VET01-S2 (2013a) as recently described (de Jong et al., 2014, van Hout et al., 2016). Seventeen different antimicrobial compounds, representing nine antimicrobial classes, were tested across a range of two-fold step dilutions (Table 1). Quality controls were included according to CLSI recommendations VET01-A4 (2013b) and VET01-S2 (2013a); reference strains of *Enterococcus fecalis* (ATCC 29212), *Staphylococcus aureus* (ATCC 29213), and *Streptococcus pneumoniae* (ATCC 49619) were used for this purpose.

**Table 1:** MIC distribution for all *S. suis* isolates in the study between 2009 and 2014. (For interpretation of the references to colour in the Table, the reader is referred to the web version of this article.)

Note: White cells indicate the dilution range tested. Values in the grey indicate MIC values over the highest concentration in the tested range. Green and red vertical lines respectively describe the sensitive and resistant clinical breakpoints recommended by the CLSI, 2013a, CLSI, 2013b. Detailed information about MIC values for each subset of isolates is available in the supplementary tables.

^*^Amoxicillin/Clavulanate combination was tested in a concentration ratio of 2:1. MIC values in the table represent Amoxicillin concentrations.

^**^Trimethoprim/Sulfamethoxazole combination was tested in a concentration ratio of 1:2. MIC values in the table represent Sulfamethoxazole concentrations.

### 2.3 Data analysis

MIC distributions for CC and NCC isolates were analysed separately for 2009–2011 and 2013–2014, using the following methods.

#### 2.3.1 MIC value distribution and epidemiological cut-off values (ECOFF)

MIC distributions were evaluated for the presence of one or more clusters. Distributions were classed as unimodal where MIC values were spread surrounding a central value, or median, in one “bell-shaped” cluster and multimodal when two or more clusters represented multiple phenotypic groups.

The ECOFF values were defined as the highest MIC value of the wild-type (WT) isolates distribution and isolates with MIC values over the ECOFF are considered non-wild type (NWT). The WT cluster includes isolates that are devoid of phenotypically detectable resistance mechanisms, while NWT isolates are spread in a range of higher MIC values as resistance mechanism are expressed. (Pfaller et al., 2011, Kahlmeter, 2015). Visual inspection of MIC distribution is a common method to determine the value of the ECOFF (Kahlmeter, 2014) and is simpler than other methods involving statistics (Turnidge et al., 2006, Kronvall, 2010). This study supported the visual assessment with a statistical analysis using mixtures of one to four normal distributions fitted to the log-transformed MIC values by maximum likelihood, and compared their support using Akaike’s Information Criterion (AIC) to describe MIC distribution and identify ECOFF values. Final ECOFF values were confirmed taking account recent literature including *S. suis* ECOFF values (Callens et al., 2013), MIC distribution (de Jong et al., 2014, van Hout et al., 2016) and the EUCAST antimicrobial resistance database (http://mic.eucast.org)

Prevalence of WT and NWT for the different antimicrobials and subsets were compared using Pearson’s chi-square test, or Fisher’s exact test for those tests with expected frequencies below 5.

#### 2.3.2 Classification using clinical breakpoints

Isolates were classified as sensitive, resistant or intermediate according to the CLSI clinical breakpoints (2013b) for the *Streptococcus* spp. for those antimicrobials with available recommendations: amoxicillin/clavulanate (AMC), penicillin, ceftiofur, tetracycline, enrofloxacin, trimethoprim/sulfamethoxazole (TMPS), erythromycin and florfenicol. Differences in prevalence of resistant isolates within the four subsets were assessed using the Pearson’s chi-squared test or Fisher’s exact test when expected frequency values were below 5.

#### 2.3.3 Differences in MIC values (including MIC_50_) between specific subsets

Changes in the characteristics of the MIC distributions for each subset over time, or between CC and NCC subsets, were identified by analysing each subset of MIC values for each antimicrobial with the Mann-Whitney-Wilcoxon test. For bimodal and multimodal MIC distributions Mann-Whitney-Wilcoxon test and MIC_50_, calculated as the median, were individually computed for WT and NWT clusters. MIC_50_ and the MIC_90_ were calculated as the MIC that inhibited the growth of 50 and 90%, respectively, of the isolates in a subset or cluster.

#### 2.3.4 Number of NWT phenotypes per isolate for different antimicrobial classes

The 17 antimicrobials were classified into nine classes (Table 2). If an isolate presented a NWT phenotype for an antimicrobial in the class, it was considered as NWT for the whole class. Differences in prevalence were assessed using the Pearson’s chi-squared test and Fisher’s exact test as an alternative when computing expected values below 5.

**Table 2:** MIC distribution patterns, ECOFFs and range of the different clusters in the whole data collection.

Class (subclass)	Antimicrobial	MIC distribution pattern	ECOFF (μg/mL)	Wild type cluster (μg/mL)	Non wild type clusters (μg/mL)
Beta lactams					
(Penams)	Amoxicillin	Unknown	0.12^*^	≤0.03–0.12	0.5–4
	AMC	Unknown	0.25^*^	≤0.03–0.25	0.5–4
	Penicillin	Unknown	0.03^*^	≤0.03	0.06–4
(Cephalosporins)	Ceftiofur	Unimodal	NA	0.03–8	NA
	Cefquinome	Unimodal	NA	0.002–0.5	NA
Amphenicols	Florfenicol	Unimodal	NA	0.5–4	NA
Pleuromutilins	Tiamulin	Multi-modal	2	0.06 – >2	4 – >64
Tetracyclines	Tetracycline	Multi-modal	4	0.25–4	16 – >128
	Doxycycline	Multi-modal	0.5	0.06–0.5	4–64
	Erythromycin	Multi-modal	0.12	0.015–0.12	2 – >32
Macrolides	Tylosin	Multi-modal	4	0.25–4	128 – >256
	Tilmicosin	Multi-modal	32	0.5–32	>128
Lincosamides	Lincomycin	Multi-modal	0.25^*^	0.06–0.25	0.5 – >128
Aminocyclitol	Spectinomycin	Multi-modal	64	4–64	≥512
Fluoroquinolones	Enrofloxacin	Multi-modal	NA	NA	0.12–4 and >8
	Marbofloxacin	Multi-modal	0.015	0.015	0.25–2 and 16
Potentiated sulphonamides	TMPS	Multi-modal	0.12^*^	0.015–0.12	0.25 – >32

Note: Antibiotics including florfenicol, ceftiofur and cefquinome presented a unimodal distribution therefore ECOFF values were not set and NWT cluster were not considered. Wild-type cluster was not considered in the case of enrofloxacin.

AMC: Amoxicillin/Clavulanate. TMPS: Trimethoprim/Sulfamethoxazole. NA: Not applicable (ECOFF were unable to be defined in this sample set).

^*^Tentative cut-off for the most susceptible cluster.

Data were analysed with the statistical software R version 3.3.1 (R Core team, Vienna, Austria) and SPSS (IBM Corp, Armonk, NY, USA). A significance level of 95% (p value of 0.05) was selected for all statistical tests. Multiple analysis testing correction was not considered strictly necessary in this study as it described findings from surveys without any specific key hypothesis (Bender and Lange, 2001) consequently the authors have designated these results as exploratory.

## 3 Results

Frequencies of MIC values for the different antimicrobials were tabulated separately for each of the four sample subsets and the combined collection (Table 1, MIC values detailed for each subset on Supplementary Table S1a–d), along with MIC_50_ values, MIC_90_ values and the percentage of sensitive (S), intermediate (I) or resistant (R) isolates where CLSI breakpoint values were available.

### 3.1 MIC value distribution and ECOFF values

The MIC distribution patterns, ECOFF values and range of the different clusters for each antimicrobial are shown in Table 2. Unimodal distributions were observed for florfenicol, ceftiofur and cefquinome (Supplementary Fig. S1a–q). MIC distributions were multimodal in the case of tetracycline, doxycycline, erythromycin, tilmicosin, tylosin, lincomycin, tiamulin, spectinomycin and marbofloxacin. For TMPS a complex distribution pattern was produced by apparent overlapping of several phenotypic clusters. MIC distribution patterns for amoxicillin, AMC, and penicillin were only partially characterized because more than 75% of the isolates were susceptible to the minimum antimicrobial dilution tested (0.003 μg/mL) (Supplementary Fig. S1a–c).

### 3.2 Differences in antimicrobial resistance between CC and NCC isolates

The percentage of isolates with a NWT phenotype was significantly higher in NCC than CC, in both 2009–2011 and 2013–2014 collections, for penicillin, tiamulin, and TMPS (Table 3). However, this pattern was not consistent for all antimicrobials, and CC isolates in 2013–14 presented a significantly higher NWT prevalence than NCC in the 2013–2014 collection for spectinomycin, lincomycin, tylosin, erythromycin and tilmicosin (Table 3). It is important to note, though, that the comparison of CC and NCC isolates from 2013 to 2014 was based upon an NCC collection obtained from a geographically restricted subset of the original 2009–2011 NCC population source.

**Table 3:** Non wild-type prevalence (%) for the different antimicrobials depending on origin (clinical (CC) or non-clinical (NCC)) and the period of collection (2009–2011 or 2013–2014), with indication of significant differences between the groups when comparing the different collections.

	Prevalence per group (%)				Pearson’s Chi-squared test*			
	2009/11		2013/14		2009/11 versus 2013/14		CC versus NCC	
	CC n = 93	NCC n = 66	CC n = 117	NCC n = 129	In CC	In NCC	In 2009/11	In 2013/14
Amoxicillin	1.1	3.0	0	2.3	ns	ns	ns	ns
AMC	1.1	3.0	0	2.3	ns	ns	ns	ns
Penicillin	9.8	34.8	14.5	38.0	ns	ns	P = 0.004	P = 0.0007
Cefquinome	NA	NA	NA	NA	NA	NA	NA	NA
Ceftiofur	NA	NA	NA	NA	NA	NA	NA	NA
Doxycycline	71.0	81.8	81.2	84.5	ns	ns	ns	ns
Tetracycline	71.0	81.8	81.2	84.5	ns	ns	ns	ns
Tiamulin	10.8	45.5	23.1	44.2	P = 0.020	ns	P = 0.000001	P = 0.0005
Enrofloxacin	100	100	100	100	ns	ns	ns	ns
Marbofloxacin	88.2	86.4	100	100	P = 0.0001	P = 0.00005	ns	ns
TMPS	17.2	46.5	44.4	58.9	P = 0.00003	ns	P = 0.00002	P = 0.023
Tilmicosin	45.2	36.4	54.7	41.1	ns	ns	ns	P = 0.033
Tylosin	45.2	36.4	54.7	41.1	ns	ns	ns	P = 0.033
Erythromycin	45.2	39.4	54.7	41.9	ns	ns	ns	P = 0.044
Lincomycin	87.1	89.4	92.3	81.4	ns	ns	ns	P = 0.012
Spectinomycin	3.2	3.0	13.7	2.3	P = 0.013	ns	ns	P = 0.001
Florfenicol	NA	NA	NA	NA	NA	NA	NA	NA

AMC: Amoxicillin/Clavulanate. TMPS: Trimethoprim/Sulfamethoxazole. NA: Not applicable due to the unimodal distribution of MIC values.

* ns: not significant over P > 0.05. Fisher’s exact test was alternatively used when the frequency of expected values was under 5.

For those antimicrobials where a clinical breakpoint was available (CLSI, 2013b), tetracycline resistant isolates were significantly more prevalent in NCC than CC in 2009–2011 and again in 2013–2014 (NCC = 97%, CC = 77%, P < 0.01; NCC = 100%, CC = 88%, P < 0.01 respectively).

Significant differences were observed in MIC values (including MIC_50_) between CC and NCC subsets. In 2009–2011 MIC values were higher among NCC than CC isolates for tetracycline (both NWT and WT cluster), cefquinome and ceftiofur (Table 4). Higher values were also seen for doxycycline (only in the NWT cluster) and erythromycin (only in the WT cluster).

**Table 4:** Differences in MIC values (including MIC_50_, MIC_90_) between CC and NCC shown separately for 2009–2011 and 2013–2014.

Note: Only those antimicrobial clusters with significant differences are represented. See supplementary table S1a-d for absent values.

^*^Significance determined by Mann-Whitney-Wilcoxon method. In blank those antimicrobials without significant differences. In grey those MIC values that were higher than compared group.

In 2013–2014 MIC values were significantly higher for NCC isolates than CC isolates for cefquinome and ceftiofur and in the WT cluster for tetracycline, as noted in 2009–2011. MIC values were significantly higher for CC than NCC isolates in 2013–2014 for florfenicol, for the NWT clusters of doxycycline, enrofloxacin, marbofloxacin and erythromycin, and for the WT cluster of tiamulin.

### 3.3 Differences in antimicrobial resistance between 2009 and 2011 and 2013–2014

There was a trend towards increased resistance between the first (2009–2011) and second (2013–2014) period, in terms of NWT prevalence, prevalence of isolates over the CLSI clinical breakpoint and, finally, MIC values for both NCC and CC subsets.

In CC isolates, NWT prevalence significantly increased between 2009 and 2011 and 2013–2014 for marbofloxacin, tiamulin, TMPS and spectinomycin (Table 3). For the NCC isolates, the NWT prevalence significantly increased between periods only for marbofloxacin.

Increases in prevalence of clinical resistance between the two time-periods, based on CLSI breakpoints, were observed for tetracycline in the CC subset (77% of isolates resistant in 2009–2011, 88% in 2013–2014; P < 0.05). Resistance prevalence was also higher in 2013–2014 compared to 2009–2011 for TMPS among CC isolates (6% resistant in 2009–2011 versus 15% in 2013–2014; P < 0.05).

Differences in MIC values (including MIC_50_) between periods were observed when comparing MIC values within equivalent clusters of isolates based on their WT or NWT grouping (Table 5). This time-based incremental change in resistance, termed as MIC creep, consisted of a general shift of MIC values in a cluster between periods. It is graphically represented as a slight movement of the MIC distribution towards higher MIC values. MIC creep phenomena were more common in CC isolates: doxycycline MIC_50_ values in the NWT cluster for CC were higher in 2013–2014 compared to CC isolates in 2009–2011. For some antibiotics, MIC distributions for CC isolates showed significantly higher values in 2013–2014 than 2009–2011 despite similar MIC_50_ values in both periods; This was found for cefquinome, ceftiofur, the WT cluster for erythromycin, and the NWT cluster for tetracycline in CC isolates.

**Table 5:** Differences in MIC values (including MIC_50_, MIC_90_) between 2009 and 2011 and 2013–2014 collections shown separately for CC and NCC groups.

Note: Only represented those antimicrobial clusters with significant differences. See supplementary table S1a-d for absent values.

^*^Significance. *p*-values calculated by Mann-Whitney-Wilcoxon method. In blank those antimicrobials without significant differences. In grey those MIC values that were higher than compared group.

For the NCC subsets, noting previously mentioned caveats over comparability, MIC values were higher in 2009–2011 than 2013–2014 for NWT clusters of doxycycline and tetracycline (Table 5).

### 3.4 Combinations of increased resistance to multiple antimicrobials

Every isolate possessed a NWT phenotype for fluoroquinolones and a WT phenotype for amphenicols. Fourteen out of 405 isolates were categorised as WT for all the antimicrobial classes excepting fluoroquinolones. At least 56% of the isolates in every subset presented four or more NWT phenotypes for the different antimicrobial classes (Table 6). NCC isolates accumulated a higher number of NWT phenotypes than CC in both time periods reaching statistical significance when considering five or more NWT phenotypes per isolate (P < 0.05). In general, the number of multiple NWT phenotypes per isolate at class level was higher in 2013–2014 than 2009–2011. Prevalence of NWT for five or more classes in CC isolates was significantly higher in 2013–2014 than 2009–2011 (30% versus 16%; P < 0.05) but no significant differences were observed in NCC isolates between periods. A small number of isolates (12/405) were identified as NWT for all groups with the exception of cephalosporins and amphenicols, and represented 2% of CC isolates in 2009–2011 and 8% of CC isolates in 2013–2014 (Table 6).

**Table 6:** Cumulative frequency of isolates (as%) showing NWT phenotypes for multiple antimicrobial classes.

Number of NWT phenotype for different antimicrobial groups.	2009/11		2013/14		2009/11 vs 2013/14		CC vs NCC	
	CC n = 93	NCC n = 66	CC n = 117	NCC n = 129	in CC	in NCC	in 2009/11	in 2013/14
≥1	100%	100%	100%	100%	ns	ns	ns	ns
≥2	98%	95%	99%	94%	ns	ns	ns	ns
≥3	63%	88%	85%	81%	P = 0.0004	ns	P = 0.0006	ns
≥4	56%	67%	66%	77%	ns	ns	ns	ns
≥5	16%	41%	30%	45%	P = 0.020	ns	P = 0.0005	P = 0.015
≥6	6%	30%	21%	32%	P = 0.004	ns	P = 0.00006	P = 0.007
≥7	4%	15%	15%	20%	P = 0.009	ns	P = 0.017	ns
8	2%	2%	8%	0%	ns	ns	ns	P = 0.001

Note: ten groups of antimicrobials were considered beta-lactams (penams and cephalosporins), macrolides, lincosamides, tetracyclines, aminoglycosides, pleuromutilins, amphenicols, fluoroquinolones and the combination of Trimethoprim/Sulfamethoxazole. Statistics performed with Pearson’s chi-squared test or Fisher’s exact test when expected frequency were lower than 5.

ns: no significant difference (p > 0.05).

A frequent combination of NWT phenotypes for CC isolates was macrolides, tetracyclines, fluoroquinolones and lincosamides, which occurred in 48% of this collection (Supplementary Table S2).

## 4 Discussion

Despite the widespread and common involvement of *S. suis* in pig and human disease around the world, a comprehensive set of internationally accepted clinical breakpoints for antimicrobial resistance does not exist for this organism. Therefore, CLSI clinical breakpoints for closely related organisms were considered in some cases but most of the analysis was based upon the statistical comparisons of MIC value distributions between subsets of isolates, and the relative prevalence of WT and NWT susceptibility phenotypes segregated by an ECOFF. While this approach has been used for other microbial species (Pfaller et al., 2011), to our knowledge this is the first application to porcine *S. suis* isolates representing two separated time periods.

Taken together, the isolates in this study had MIC and ECOFF values in line with previous studies from other regions (Aarestrup et al., 1998, Callens et al., 2013, de Jong et al., 2014, Gurung et al., 2015, Zhang et al., 2015; van Hout et al., 2016). Data gaps in this study, which may lead to potential bias, were the lack of information on pig ages, potential for clustering of diagnostic laboratory submissions as farm level, and antimicrobial use. In addition, NCC samples collected in 2013-14 originated from a pig population smaller than the other subsets, and that could be epidemiologically related.

### 4.1 Identification of ECOFF values

ECOFFs derived using the mathematical model agreed with these obtained by subjective visual inspection in most but not all cases. Where several logarithmic steps separated the MIC values of the WT and NWT clusters then ECOFFs could easily be identified by the visual approach. However, the mathematical model was particularly useful in identifying ECOFF values where there was overlap of the WT and NWT clusters. Previously reported models barely improved visual inspection ECOFF choices (Turnidge et al., 2006, Kronvall, 2010). Although the model was designed to minimise the misclassification of isolates either side of the ECOFF biases likely exist and future models will benefit from the incorporation of genetic as well as phenotypic information. Such further refinement of mathematical methods for ECOFF identification is an important step towards standardisation of antimicrobial resistance surveillance.

### 4.2 Non-disease associated (NCC) isolates showed increased antimicrobial resistance compared to disease-associated (CC) isolates

Increased antimicrobial resistance was found for the NCC subsets compared to the CC subsets. The same effects were found for the 2009–2011 and the 2013–2014 subsets for NWT prevalence, prevalence of resistance based on published clinical breakpoints and by comparison of MIC values. Earlier studies of differential antimicrobial susceptibility among *S. suis* isolates from diseased or healthy pigs found no difference or described an increased prevalence of the macrolide-lincomycin-streptogramin B resistance phenotype (MLS_B_) among isolates from healthy sows versus isolates from diseased slaughter pigs (Zhang et al., 2015). More recently different resistance profiles for isolates from healthy or diseased slaughter pigs in Korea were described but statistically significant differences were not reported (Gurung et al., 2015).

Correlations between serotype, as a proxy for disease association, and antimicrobial resistance have previously been reported (Aarestrup et al., 1998, Wisselink et al., 2006) however those comparisons only considered serotyped disease-associated isolates. More recently, genome-wide association studies of large populations of disease-associated and non-disease associated *S. suis* revealed enormous diversity and recombination among isolates, and highlighted a significantly smaller genome size for disease-associated isolates (Weinert et al., 2015) and very low prevalence of disease-associated genomotypes of *S. suis* in the upper respiratory tract of healthy pigs. Evidence from other microbial contexts indicates that antimicrobial resistance, while conferring selective protection, can carry a broader biological cost (Andersson, 2006) that might impact on competitive fitness in ecological niches such as the upper respiratory tract. Ongoing investigation of the genomic basis for observed antimicrobial resistance phenotypes in this current collection will shed new light on the potential role of non-disease associated isolates as reservoirs for horizontally transmissible antimicrobial resistance genes. It should also be noted that isolates categorised as non-clinical in this study might, under conditions of reduced immunity, be able to escape from the upper respiratory tract to cause systemic disease. However, at the level of clinical veterinary practice and national surveillance for trends in antimicrobial resistance, this finding emphasises the importance of considering the clinical history of isolates when interpreting antimicrobial susceptibility test data.

### 4.3 Antimicrobial resistance increased in S. suis between both periods

The data showed a general trend of higher resistance between 2009 and 2011 and 2013–2014, in agreement with previous European studies of *S. suis* antimicrobial resistance conducted over recent years (Varela et al., 2013, van Hout et al., 2016). The change was particularly apparent among CC isolates for tiamulin, marbofloxacin, TMPS, spectinomycin, tetracyclines, cephalosporins and macrolides. All of these antimicrobials were used in the English pig industry before the samples used in this study were collected.

Voluntary prescribing guidance published by the UK’s Pig Veterinary Society places fluoroquinolones and 3rd or 4th generation cephalosporins (ceftiofur and cefquinome) in a category of antimicrobials intended for use only as a last resort and supported by laboratory sensitivity tests. The prevalence of NWT phenotype for marbofloxacin among CC and NCC isolates increased to 100% between 2009 and 2011 and 2013–2014. Isolates which were WT for marbofloxacin in 2009–2011 were found to have a NWT phenotype for enrofloxacin, another fluoroquinolone. A decrease in fluoroquinolone susceptibility in *S. suis* and *S. pneumoniae* has been described as a stepwise process in which first-step mutations had a preferential target in genes encoding elements of topoisomerase IV (*parC, parE*) or DNA gyrase (*gyrA*) for a reduced number of fluoroquinolones. In a second step, more amino acid substitutions are accumulated, also affecting repression of an ABC transporter efflux pump so resistance is significantly increased, conferring resistance to additional fluoroquinolone types (Escudero et al., 2011). Therefore, surveillance programs that monitor for low-level resistance against more than one fluoroquinolone have merit in detecting early and low, but progressive, resistance increase against this important class of antimicrobials.

Statistically significant but subtle increases in resistance were found for ceftiofur and cefquinome between the first and second time-periods. Comparison of MIC values showed an overall increase, or creep, between periods for both antimicrobials; although only a marginal change in MIC_50_ was found there was a shift in overall MIC distributions. The prevalence of resistant isolates according to the CLSI breakpoint for ceftiofur showed no significant change between 2009 and 2011 and 2013–2014 highlighting the potential limitations of surveillance based on clinical breakpoints for monitoring antimicrobial susceptibility trends.

Beta lactam antimicrobials other than cephalosporins, and especially penams, are considered as a first option in the UK to treat *S. suis-*related diseases. Indeed, penicillin-resistant isolates have been increasingly detected in recent years among pig isolates from European and Asian countries (Zhang et al., 2008, Callens et al., 2013). In this study, there was no evidence of any statistically significant change in resistance to penicillin, amoxicillin or amoxicillin/clavulanate. This finding concurs with those of routine surveillance reported by APHA, which describes a small number of penicillin-resistant clinical and non-clinical *S. suis* isolates since 2009 with no evidence of an increasing trend in penicillin MICs undertaken periodically on disease-associated *S. suis* isolates (APHA, 2015).

### 4.4 Combinations of increased resistance to multiple antimicrobials have become more prevalent and complex between periods

The carriage by *S. suis* of multiple resistance determinants is already well described (Chen et al., 2013) but the finding of increased prevalence and diversity of this phenomenon between both periods and also between subsets of non-disease associated NCC versus disease-associated CC origin was novel. Although the clinical implications for these resistances may not yet be fully apparent in veterinary practice as clinical breakpoints may not yet have been exceeded, there was a clear indication of ongoing and progressively increasing resistance for multiple antimicrobials. These findings, when combined with genomic data, may enable a better understanding of the co-selection of resistance and the impact of selective advantages and broader biological costs of antimicrobial resistance mechanisms on bacterial fitness and disease association.

Surveillance of antimicrobial resistance for endemic, and especially zoonotic, veterinary pathogens such as *S. suis* using internationally standardised methods is likely to become the focus of ever-increasing attention as steps are taken to develop more rational evidence-based approaches to antimicrobial use in food animal production. From the perspective of clinical veterinary management of disease, MIC value distributions and epidemiological cut-offs are less helpful in predicting clinical success of antibiotic treatments compared to clinical breakpoints − where such breakpoints are available. However, the power of surveillance based on MIC values and NWT prevalence lies in their prospective usefulness in early identification of changes in *S. suis* antimicrobial susceptibility and the emergence of resistant strains, as well as for monitoring the effectiveness of antimicrobial resistance control strategies. Such changes may be detected before the practical consequences of antimicrobial resistance, based on clinical breakpoint data, become apparent.

## 5 Conclusion

Large-scale studies of the antimicrobial resistance phenotype of bacterial isolates are necessary to assign ECOFF values and clinical veterinary breakpoints. This study used standardised methods and carefully curated groups of isolates to identify significant shifts in antimicrobial resistance phenotypes of *S. suis*, isolated from pigs in England, not only between two time periods but also between isolates with known disease or non-disease associated background. Nonetheless, we highlighted relevant data gaps and potential biases in our sample set which reflect the challenge of composing sufficiently large collections, with detailed phenotypic data, for statistically meaningful analysis of this important zoonotic pig pathogen.

Surveys such as this represent a prerequisite step to better understanding of the connections between genotype, phenotype and clinical antimicrobial responses. Outputs from such surveys, when combined with subsequent genome sequencing, represent a crucial step towards comprehensive understanding of the genomic basis for the biology, evolution and management of antimicrobial resistance. Such combined approaches are likely to be valuable in optimising antimicrobial resistance surveillance programs, informing appropriate clinical antimicrobial usage, and ensuring future availability of effective antimicrobials.

## Funding

This study (BB/L003902/1) was funded by the RCUK-MOST China UK Programme on Global Priorities. JHG was funded by the Zoetis/Cambridge Senior Training Scholarship in Pig Health Management. AEM was funded by a Biotechnology and Biological Sciences Research Council grant BB/M014088/1. This work was supported by a Longer and Larger (LoLa) grant from the Biotechnology and Biological Sciences Research Council (grant numbers BB/G020744/1, BB/G019177/1, BB/G019274/1 and BB/G018553/1), the UK Department for Environment, Food and Rural Affairs and Zoetis awarded to the Bacterial Respiratory Diseases of Pigs-1 Technology (BRaDP1T) consortium.

## Conflict of interest

None.
