# Supplementary material for: Patterns of antimicrobial resistance in Streptococcus suis isolates from pigs with or without streptococcal disease in England between 2009 and 2014
Source: Vet Microbiol. 2017 Aug;207:117–24. doi: 10.1016/j.vetmic.2017.06.002 (PMC5548070; doi:10.1016/j.vetmic.2017.06.002)
Supplement: Supplementary file 1 [file mmc1.docx]

## SUPPLEMENTARY FIGURES

### Supplementary figure 1a: Amoxicillin MIC value distribution for clinical samples in 2009-2011 and 2013-2014 (i) and for non clinical samples in 2009-2011 and 2013-2014 (ii), clinical and non clinical isolates in 2009-2011 (iii) and 2013-2014 (iv).


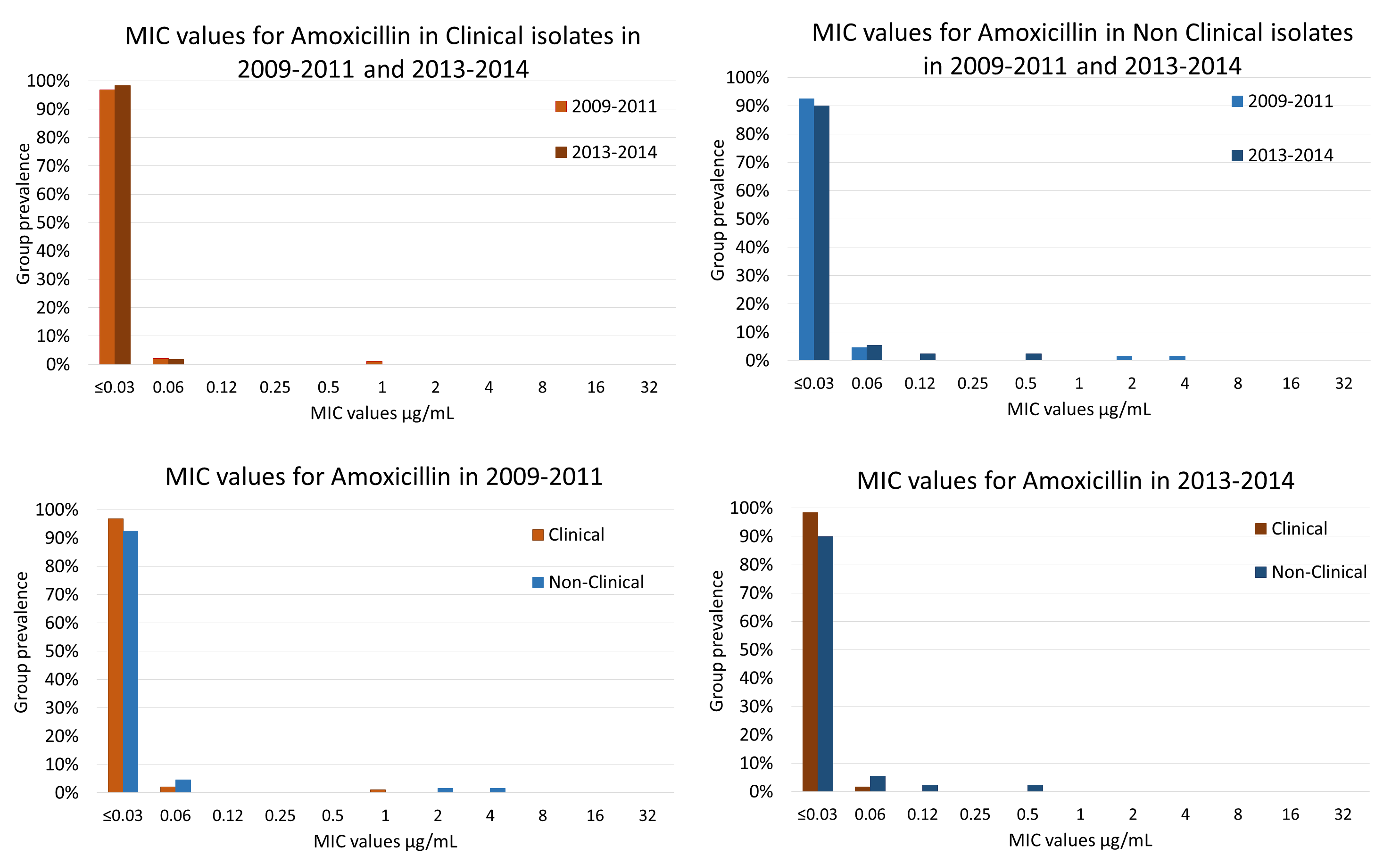


**i)**

**ii)**

**iii)**

**iv)**

### Supplementary figure 1b: Amoxicillin/Clavulanate MIC value ( for clavulanate) distribution for clinical samples in 2009-2011 and 2013-2014 (i) and for non clinical samples in 2009-2011 and 2013-2014 (ii), clinical and non clinical isolates in 2009-2011 (iii) and 2013-2014 (iv).


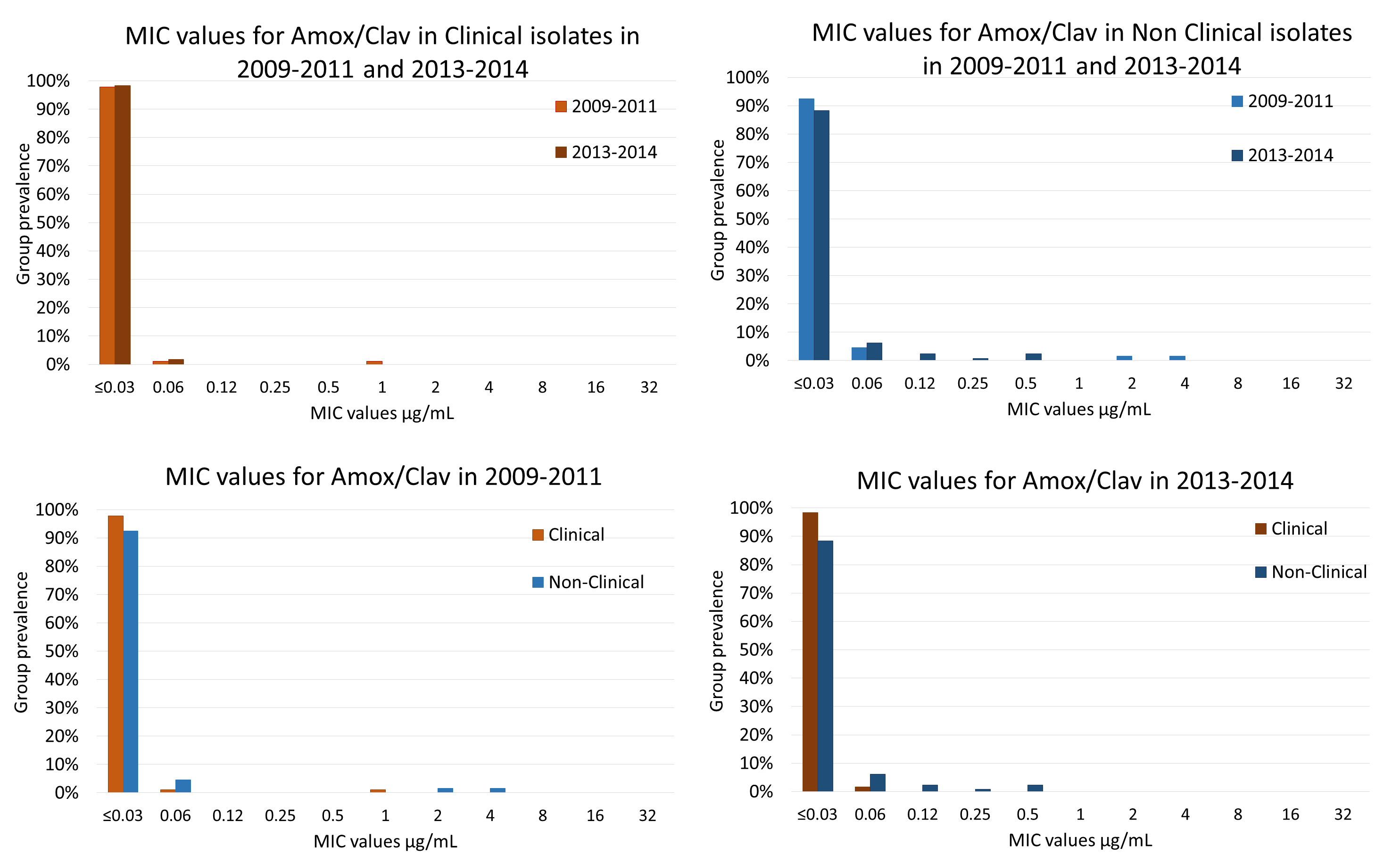


**iii)**

**iv)**

**i)**

**ii)**

### Supplementary figure 1c: Penicillin MIC value distribution for clinical samples in 2009-2011 and 2013-2014 (i) and for non clinical samples in 2009-2011 and 2013-2014 (ii), clinical and non clinical isolates in 2009-2011 (iii) and 2013-2014 (iv).


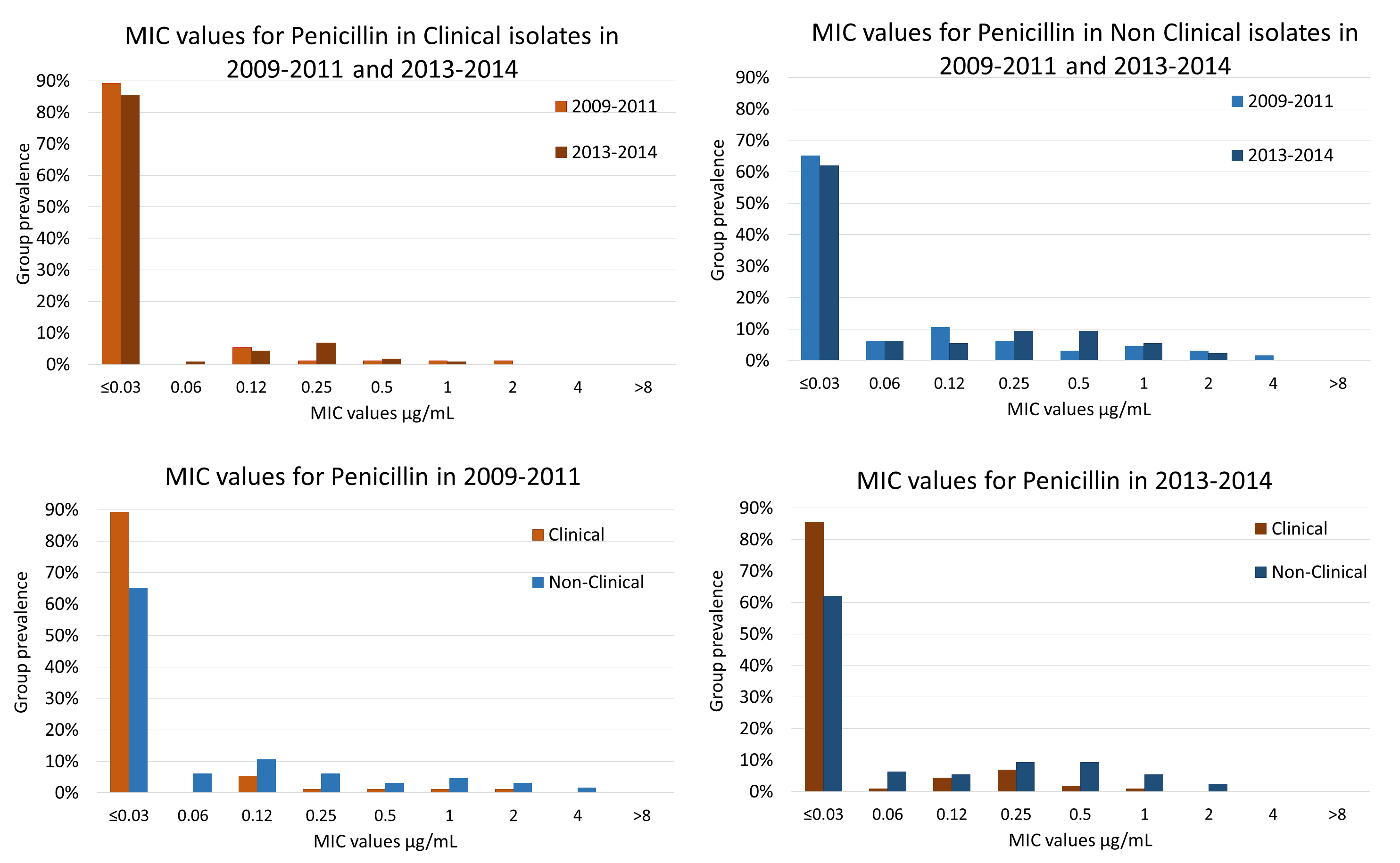


**iv)**

**iii)**

**i)**

**ii)**

### Supplementary figure 1d: Cefquinome MIC value distribution for clinical samples in 2009-2011 and 2013-2014 (i) and for non clinical samples in 2009-2011 and 2013-2014 (ii), clinical and non clinical isolates in 2009-2011 (iii) and 2013-2014 (iv).


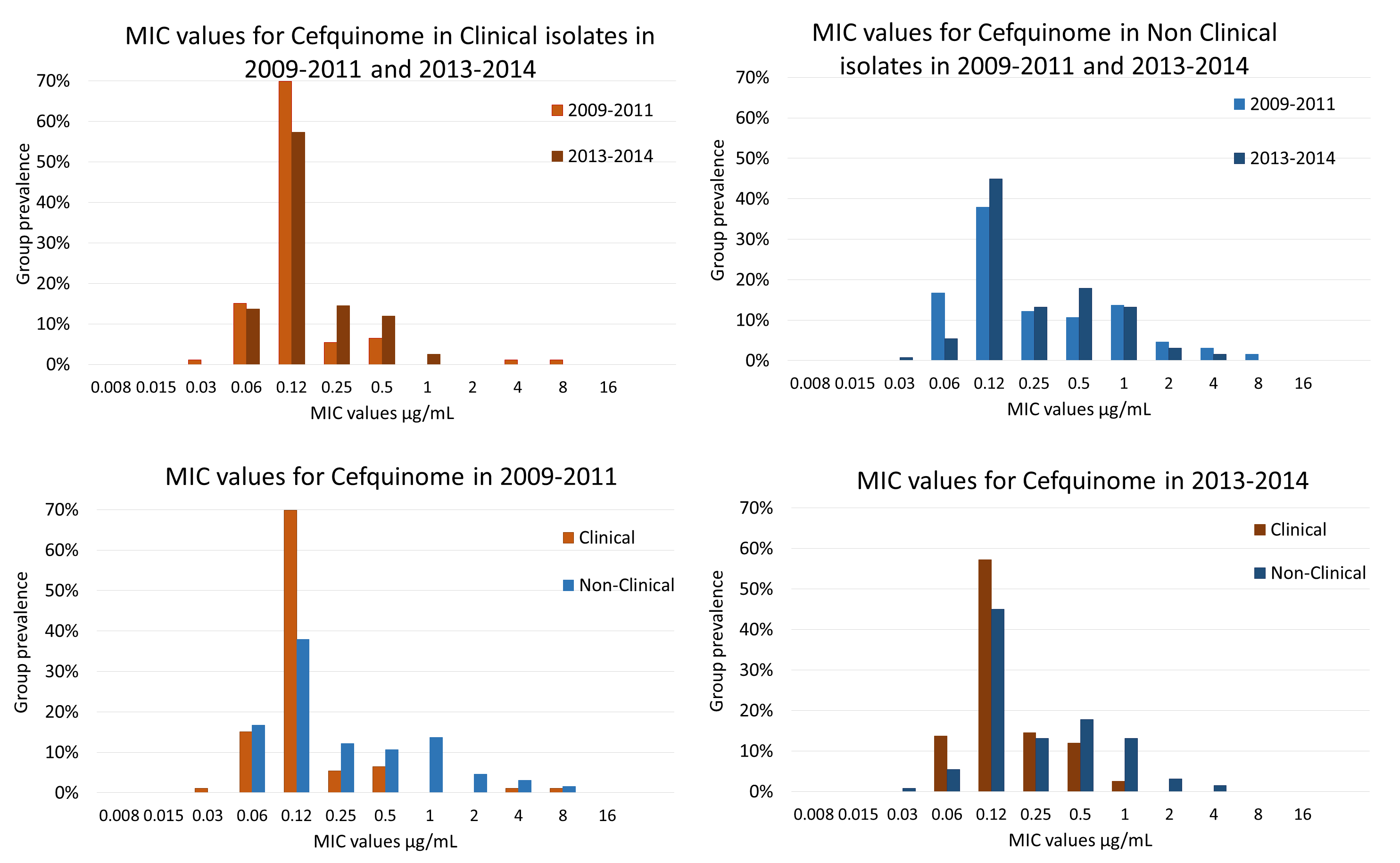


**iv)**

**iii)**

**i)**

**ii)**

### Supplementary figure 1e: Ceftiofur MIC value distribution for clinical samples in 2009-2011 and 2013-2014 (i) and for non clinical samples in 2009-2011 and 2013-2014 (ii), clinical and non clinical isolates in 2009-2011 (iii) and 2013-2014 (iv).


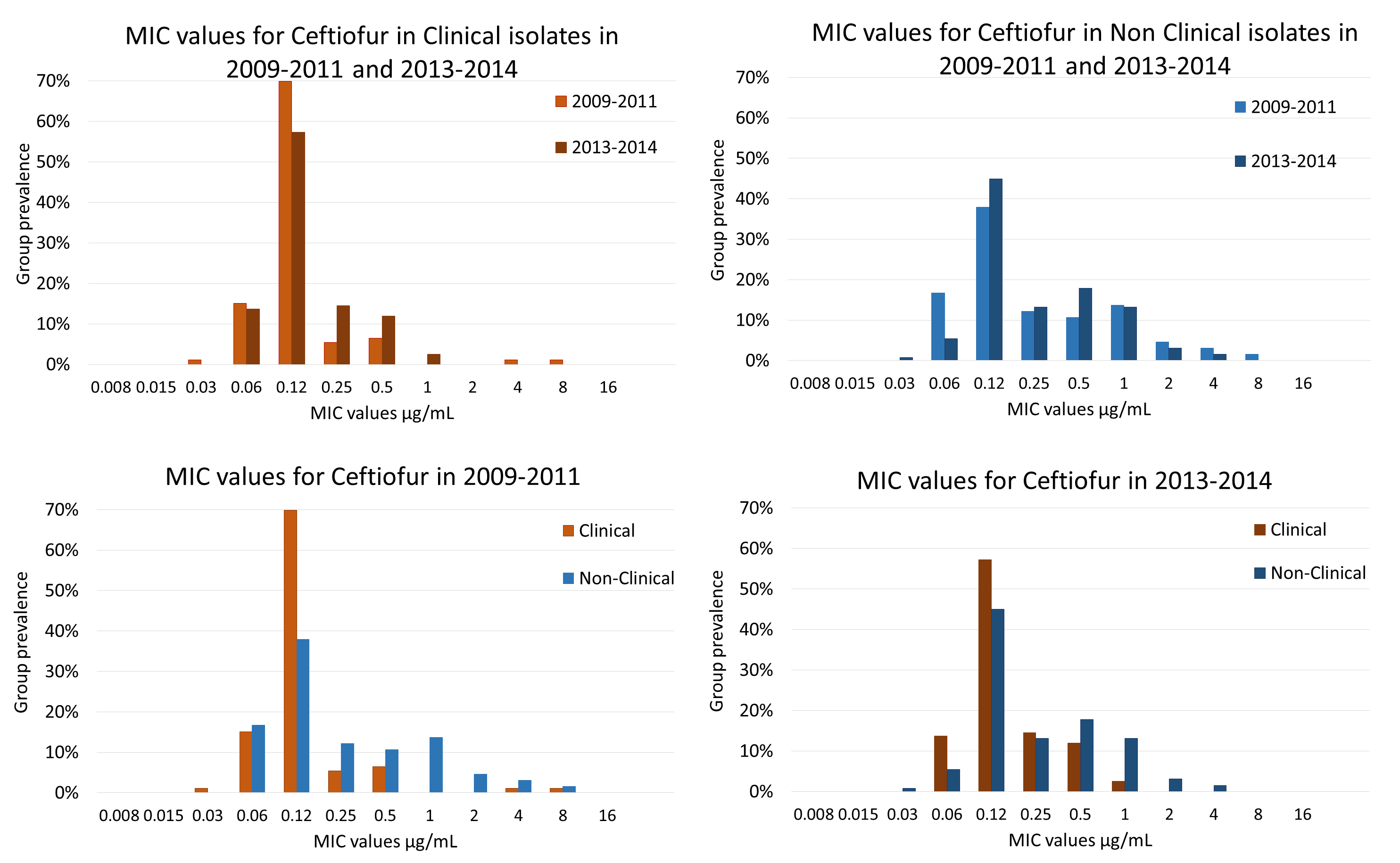


**iv)**

**iii)**

**i)**

**ii)**

### Supplementary figure 1f: Doxycycline MIC value distribution for clinical samples in 2009-2011 and 2013-2014 (i) and for non clinical samples in 2009-2011 and 2013-2014 (ii), clinical and non clinical isolates in 2009-2011 (iii) and 2013-2014 (iv).


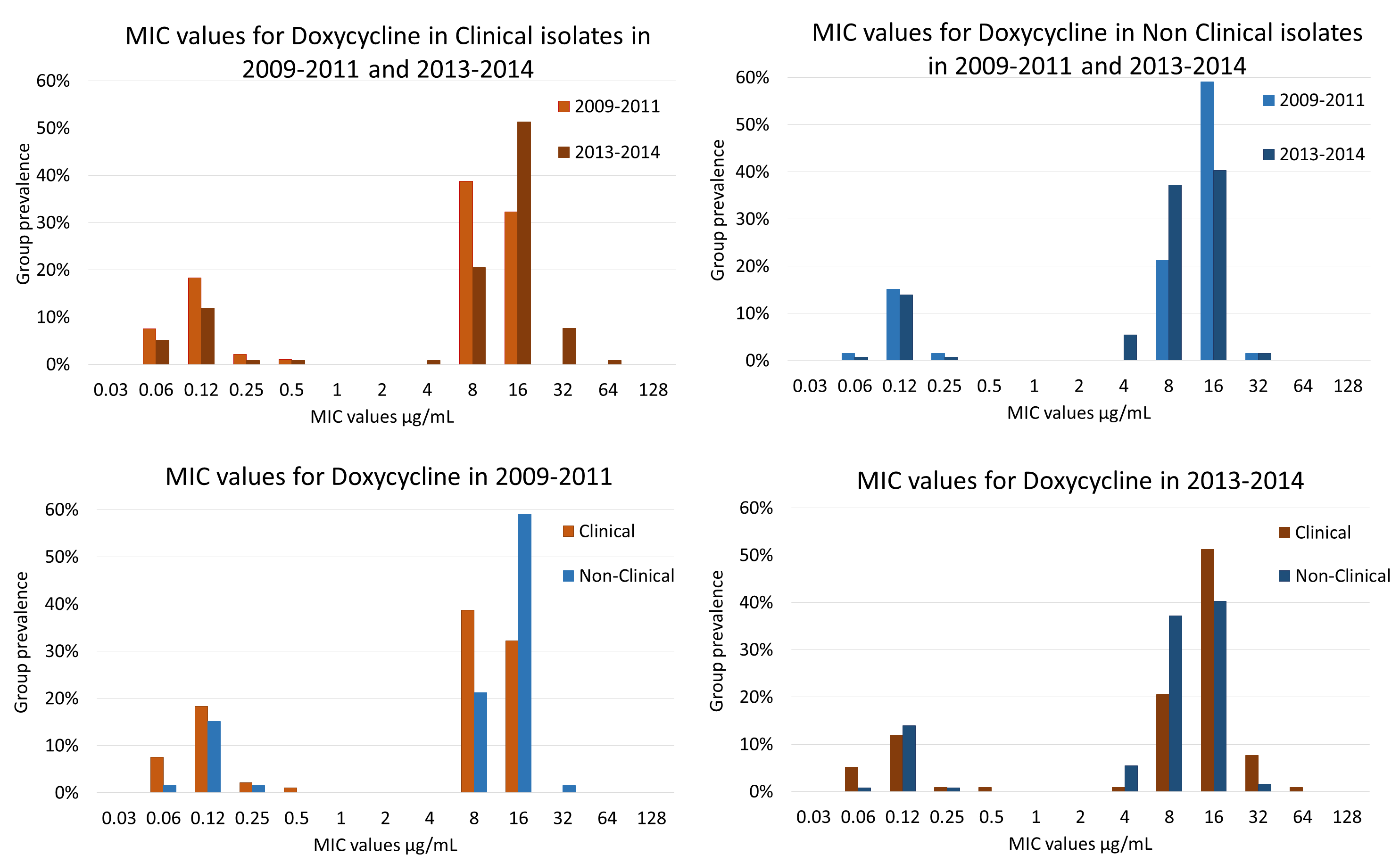


**iv)**

**iii)**

**i)**

**ii)**

### Supplementary figure 1g: Tetracycline MIC value distribution for clinical samples in 2009-2011 and 2013-2014 (i) and for non clinical samples in 2009-2011 and 2013-2014 (ii), clinical and non clinical isolates in 2009-2011 (iii) and 2013-2014 (iv).


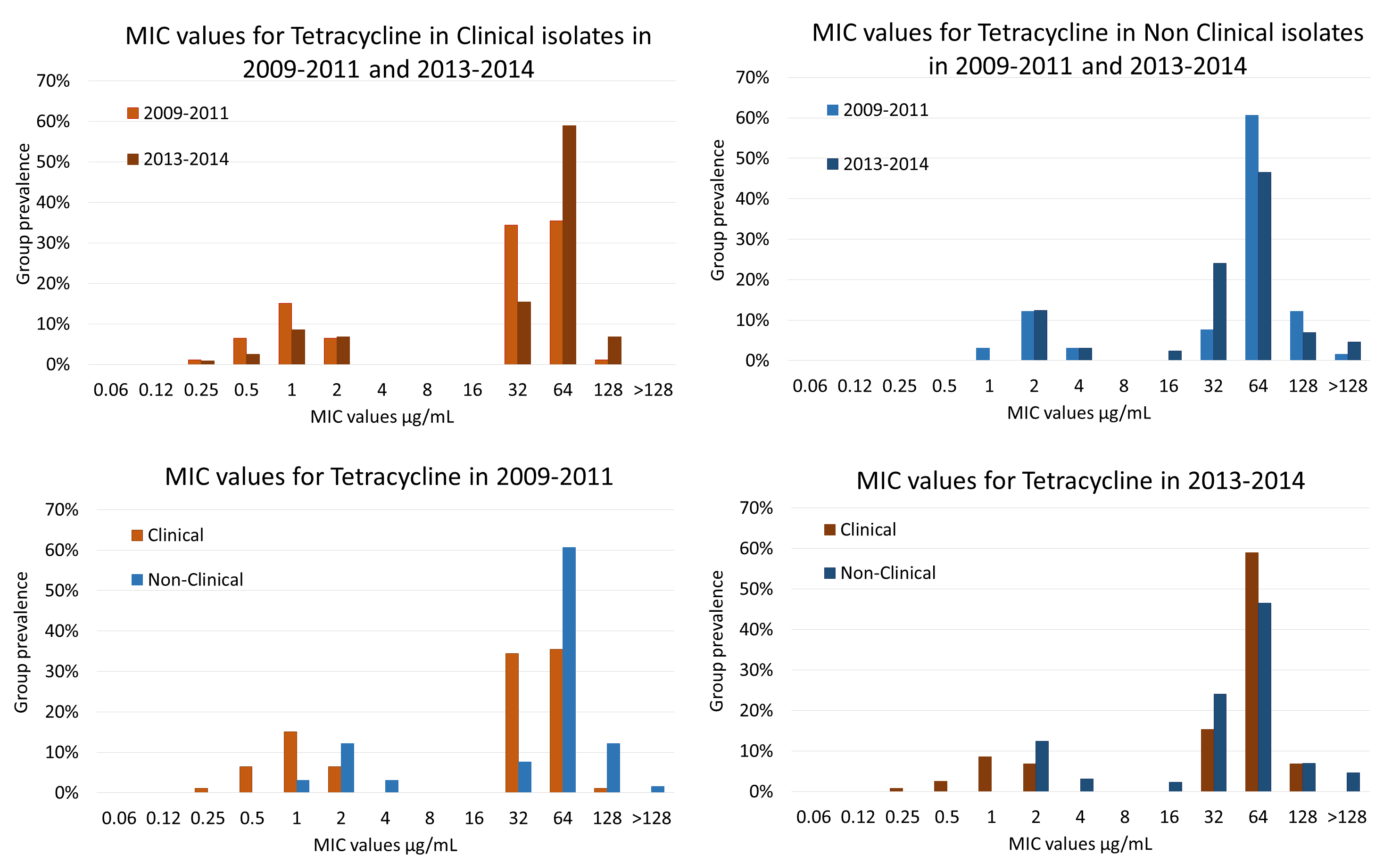


**iv)**

**iii)**

**i)**

**ii)**

### Supplementary figure 1h: Tiamulin MIC value distribution for clinical samples in 2009-2011 and 2013-2014 (i) and for non clinical samples in 2009-2011 and 2013-2014 (ii), clinical and non clinical isolates in 2009-2011 (iii) and 2013-2014 (iv).


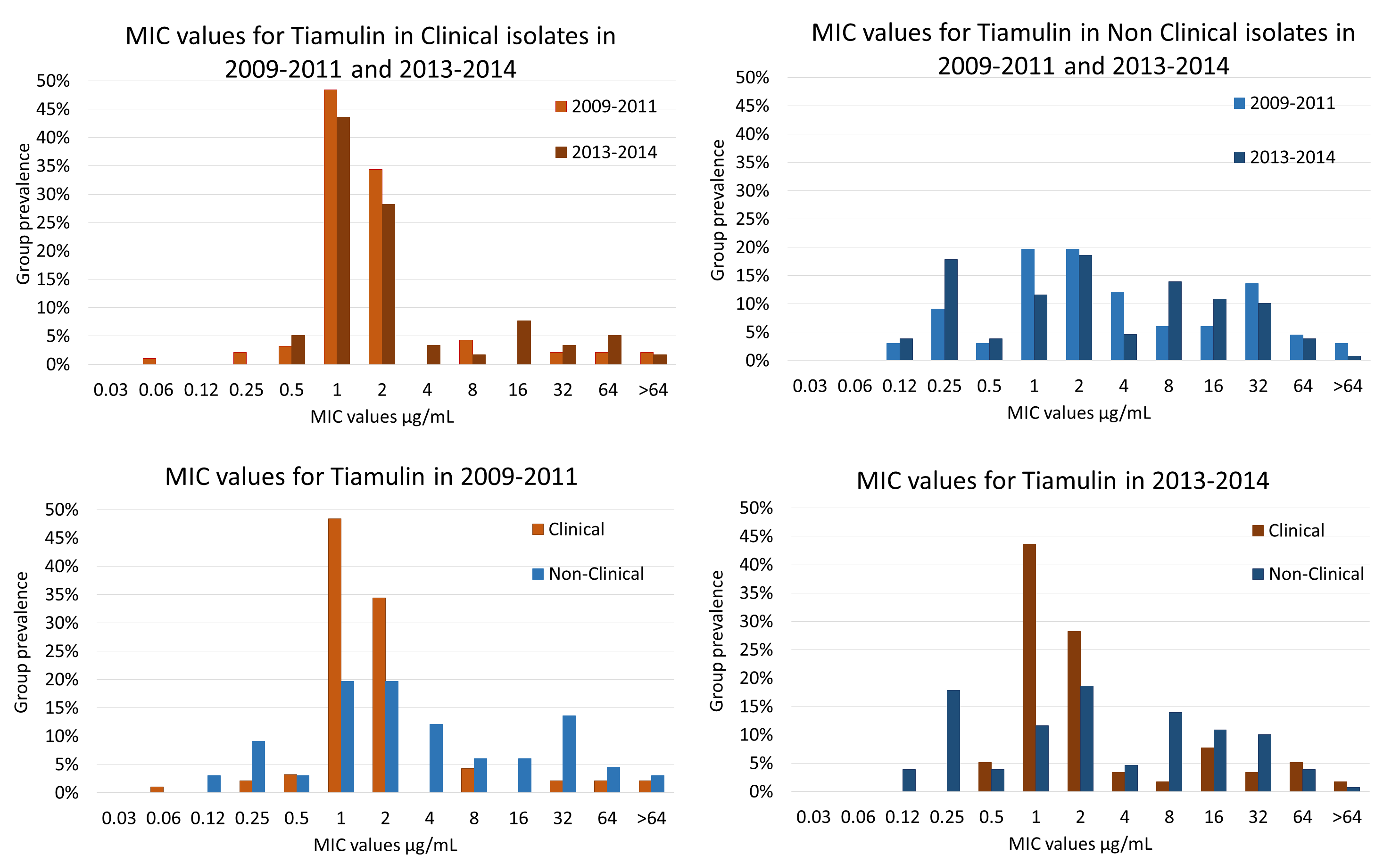


**iv)**

**iii)**

**i)**

**ii)**

### Supplementary figure 1i: Enrofloxacin MIC value distribution for clinical samples in 2009-2011 and 2013-2014 (i) and for non clinical samples in 2009-2011 and 2013-2014 (ii), clinical and non clinical isolates in 2009-2011 (iii) and 2013-2014 (iv).


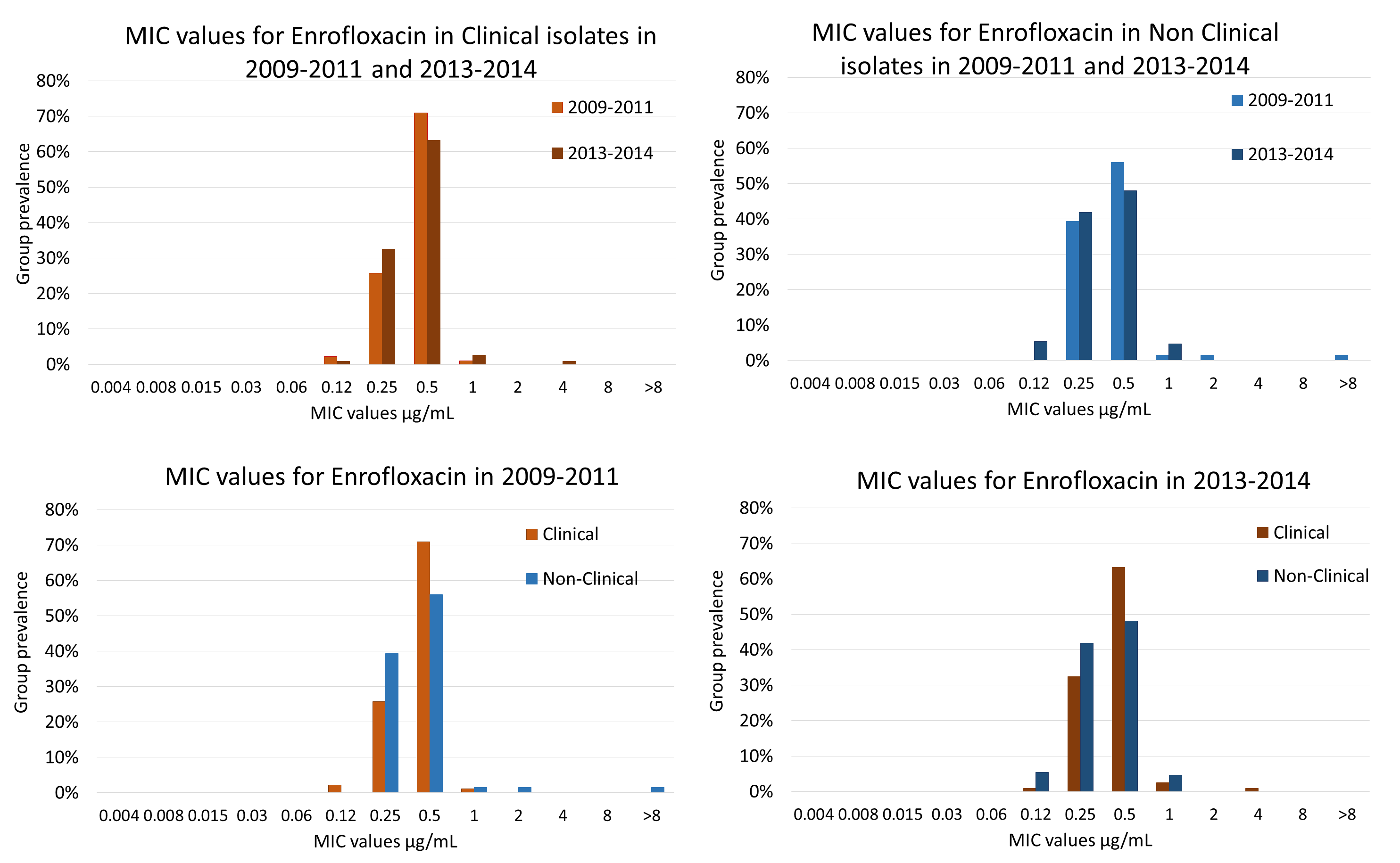


**iv)**

**iii)**

**i)**

**ii)**

### Supplementary figure 1j: Marbofloxacin MIC value distribution for clinical samples in 2009-2011 and 2013-2014 (i) and for non clinical samples in 2009-2011 and 2013-2014 (ii), clinical and non clinical isolates in 2009-2011 (iii) and 2013-2014 (iv).


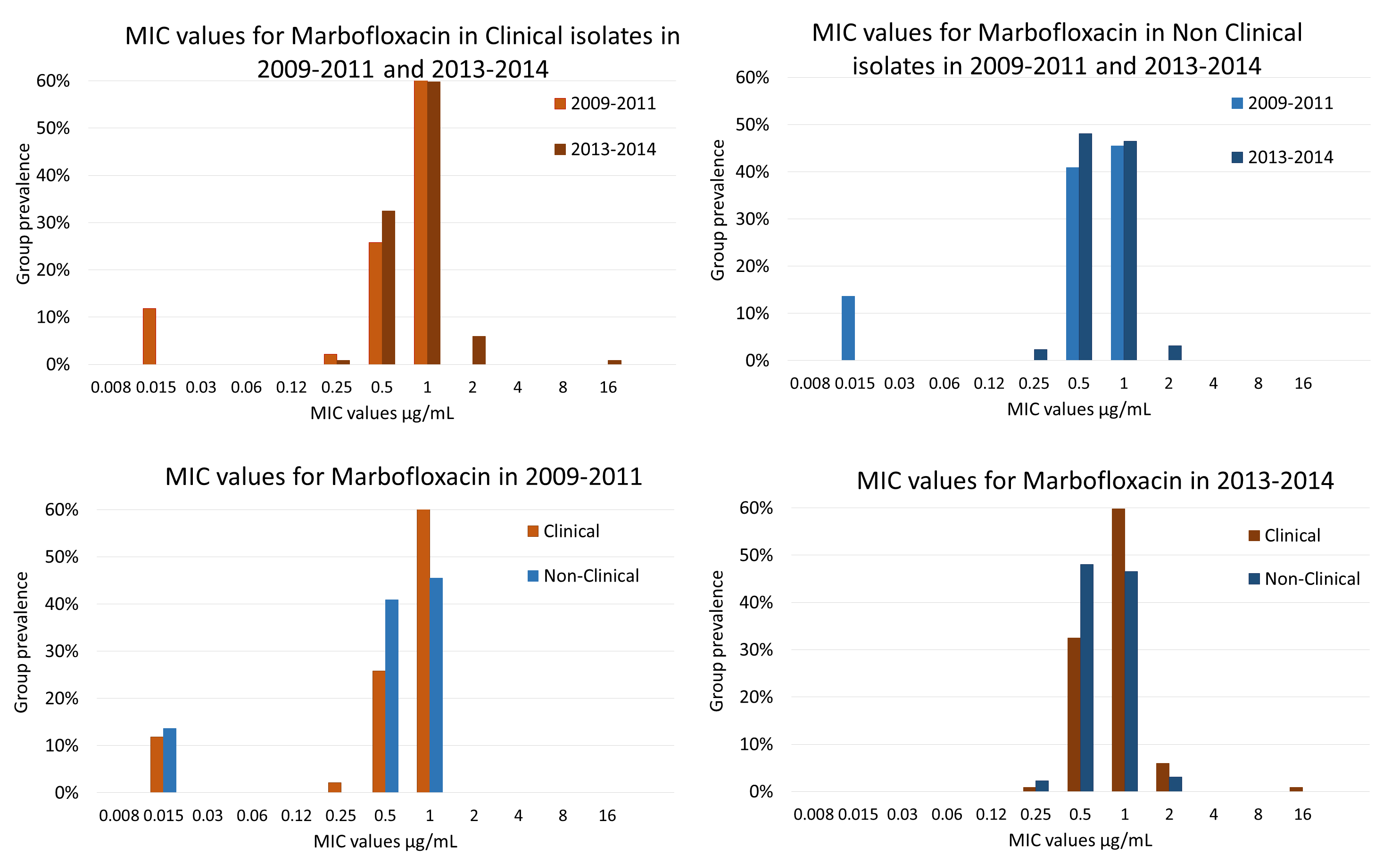


**iv)**

**iii)**

**i)**

**ii)**

### Supplementary figure 1k: Trimethoprim/sulfamethoxazole MIC value (for sulfamethoxazole) distribution for clinical samples in 2009-2011 and 2013-2014 (i) and for non clinical samples in 2009-2011 and 2013-2014 (ii), clinical and non clinical isolates in 2009-2011 (iii) and 2013-2014 (iv).


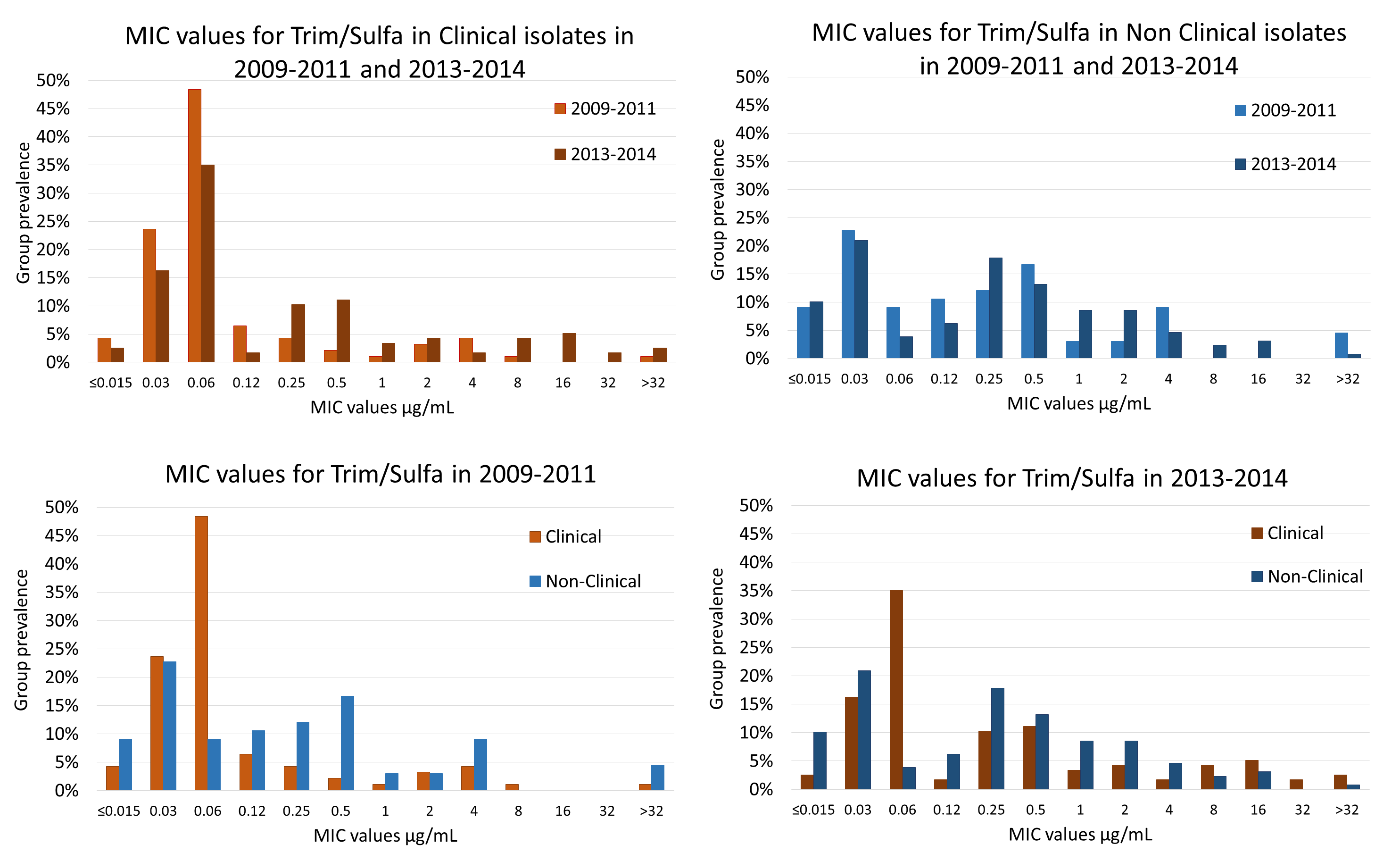


**iv)**

**iii)**

**i)**

**ii)**

### Supplementary figure 1l: Tilmicosin MIC value distribution for clinical samples in 2009-2011 and 2013-2014 (i) and for non clinical samples in 2009-2011 and 2013-2014 (ii), clinical and non clinical isolates in 2009-2011 (iii) and 2013-2014 (iv).


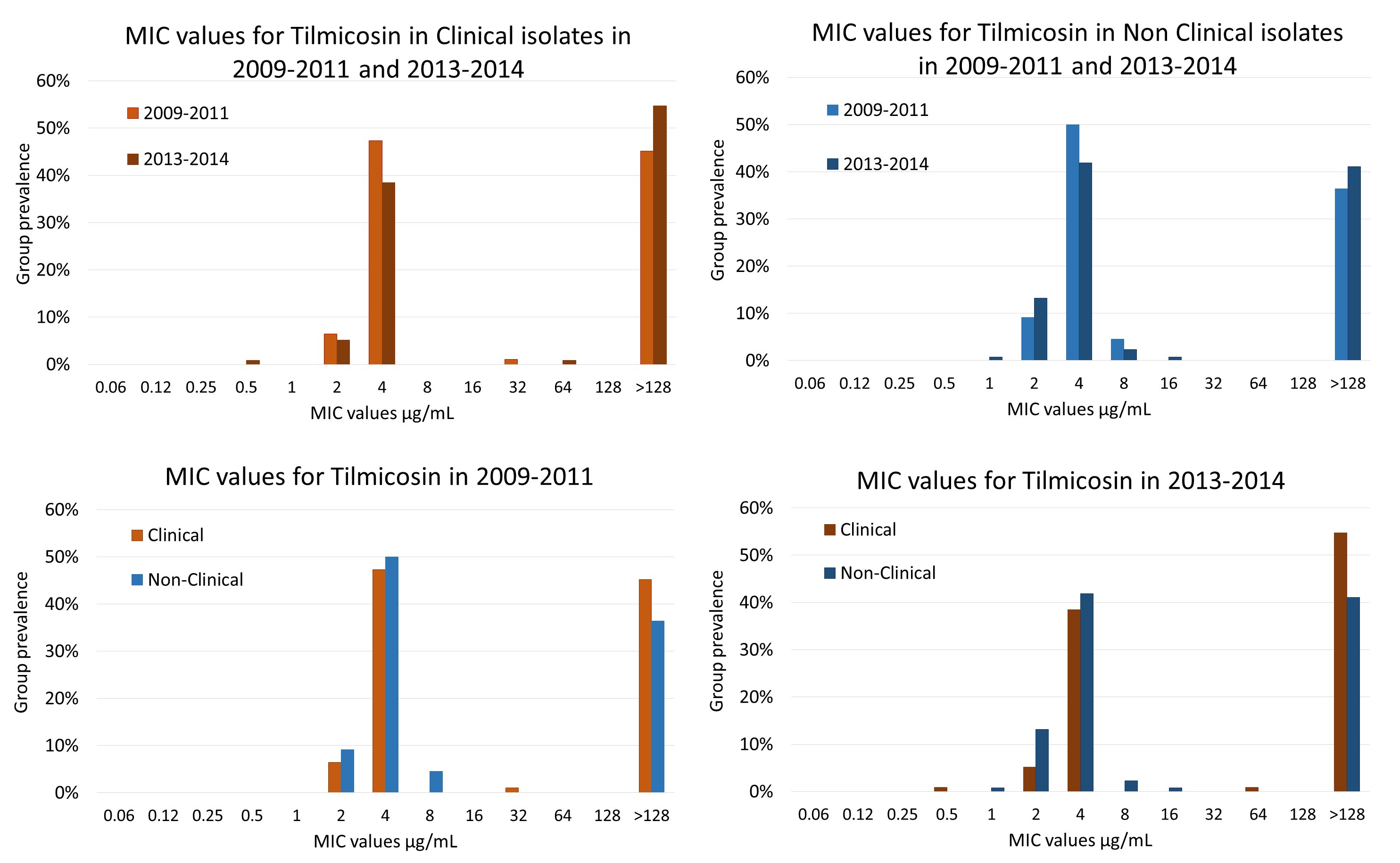


**iii)**

**iv)**

**i)**

**ii)**

### Supplementary figure 1m: Tylosin MIC value distribution for clinical samples in 2009-2011 and 2013-2014 (i) and for non clinical samples in 2009-2011 and 2013-2014 (ii), clinical and non clinical isolates in 2009-2011 (iii) and 2013-2014 (iv).


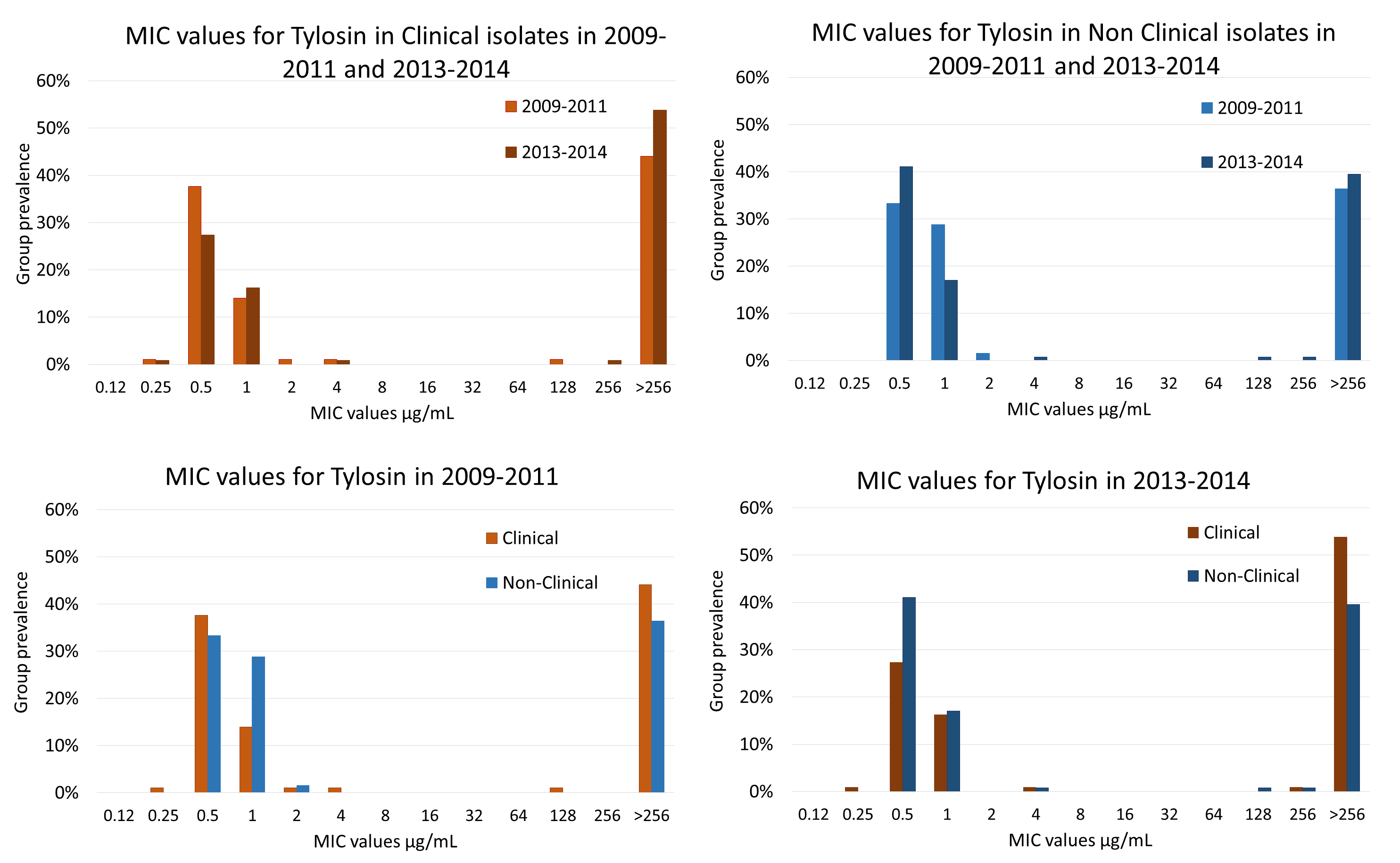


**iv)**

**iii)**

**i)**

**ii)**

### Supplementary figure 1n: Erythromycin MIC value distribution for clinical samples in 2009-2011 and 2013-2014 (i) and for non clinical samples in 2009-2011 and 2013-2014 (ii), clinical and non clinical isolates in 2009-2011 (iii) and 2013-2014 (iv).


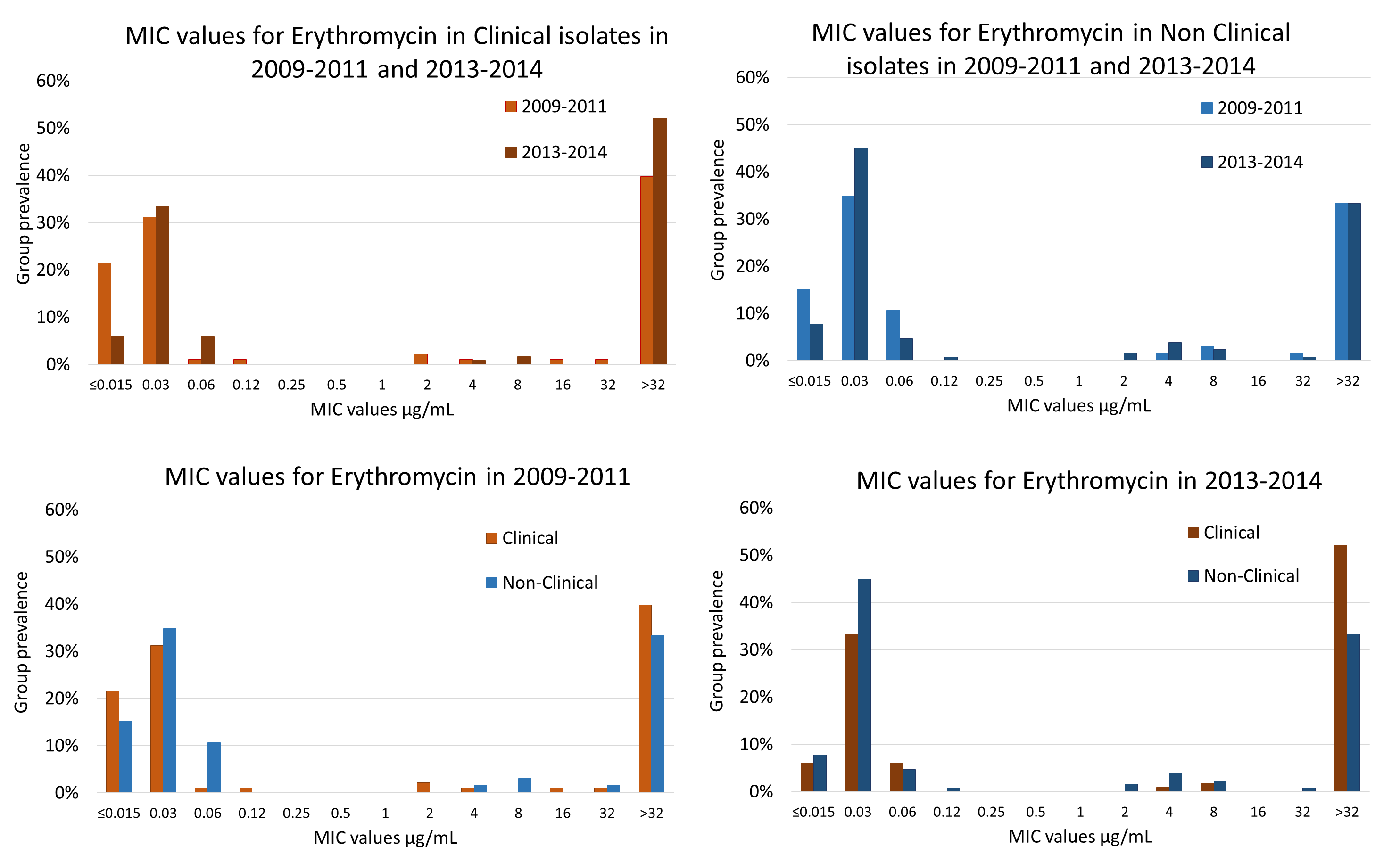


**iv)**

**iii)**

**i)**

**ii)**

### Supplementary figure 1o: Lincomycin MIC value distribution for clinical samples in 2009-2011 and 2013-2014 (i) and for non clinical samples in 2009-2011 and 2013-2014 (ii), clinical and non clinical isolates in 2009-2011 (iii) and 2013-2014 (iv).


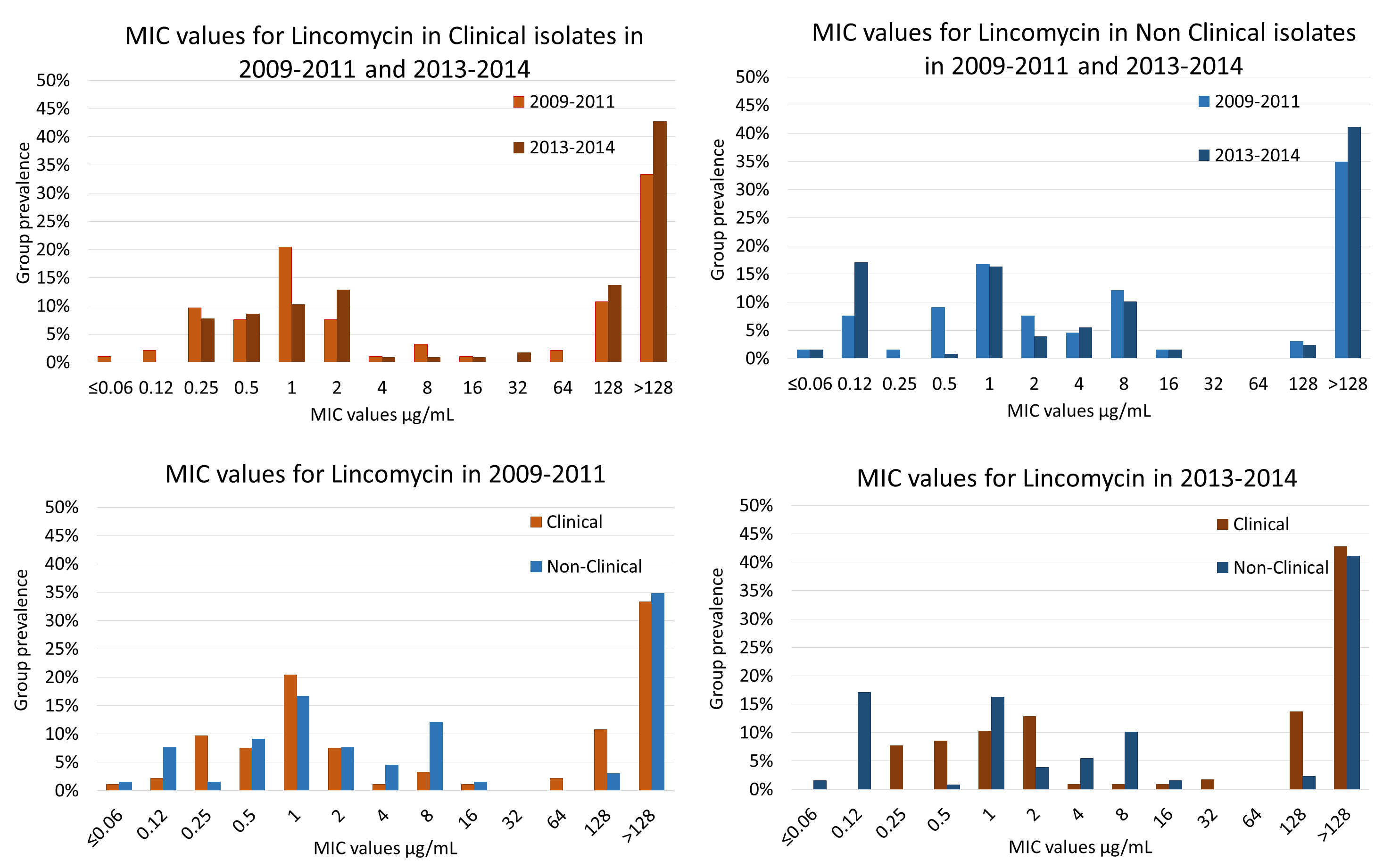


**i)**

**iv)**

**iii)**

**ii)**

### Supplementary figure 1p: Spectinomycin MIC value distribution for clinical samples in 2009-2011 and 2013-2014 (i) and for non clinical samples in 2009-2011 and 2013-2014 (ii), clinical and non clinical isolates in 2009-2011 (iii) and 2013-2014 (iv).


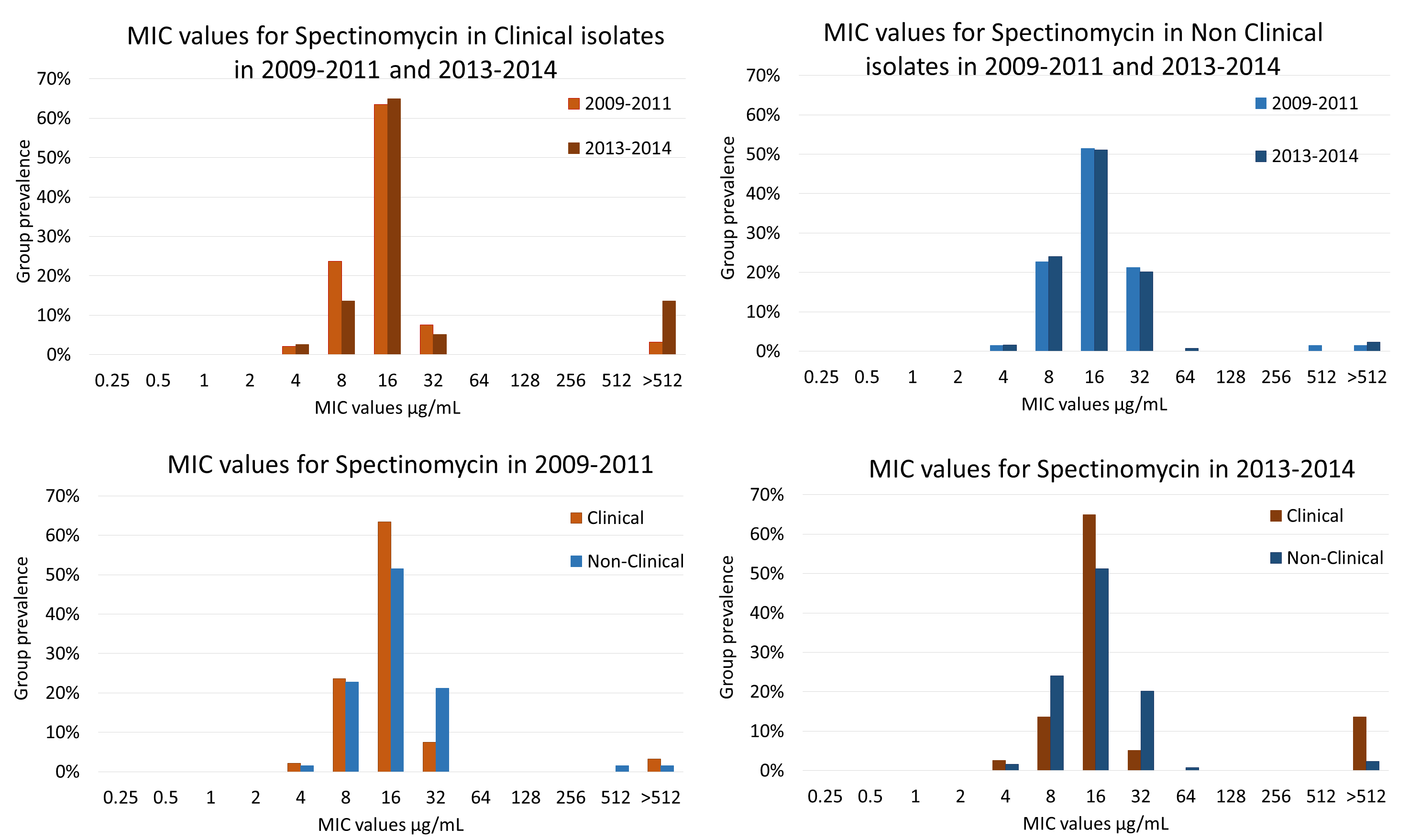


**i)**

**iv)**

**iii)**

**ii)**

### Supplementary figure 1q: Florfenicol MIC value distribution for clinical samples in 2009-2011 and 2013-2014 (i) and for non clinical samples in 2009-2011 and 2013-2014 (ii), clinical and non clinical isolates in 2009-2011 (iii) and 2013-2014 (iv).


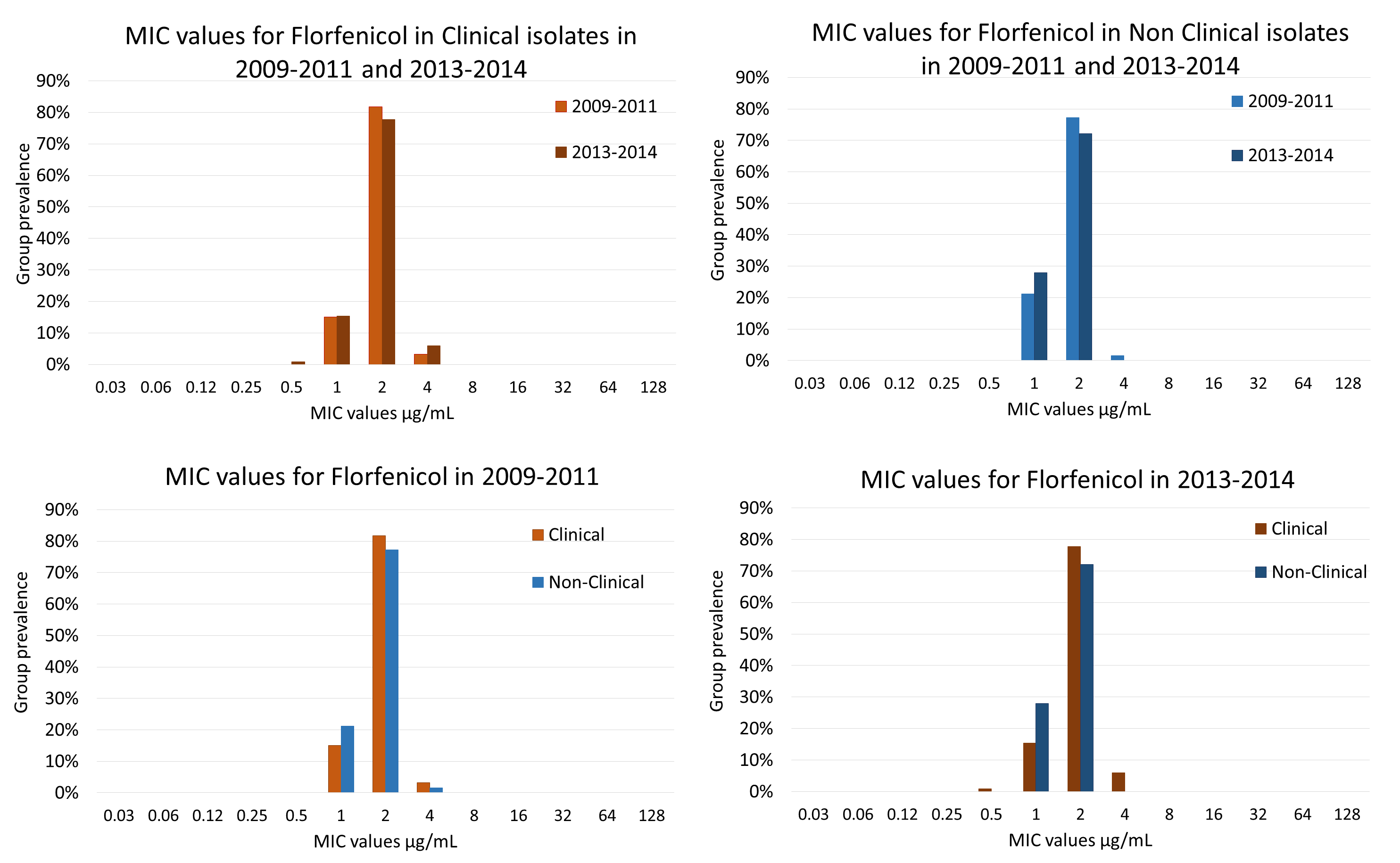


**i)**

**iv)**

**iii)**

**ii)**
