# Supplementary material for: Patterns of antimicrobial resistance in Streptococcus suis isolates from pigs with or without streptococcal disease in England between 2009 and 2014
Source: Vet Microbiol. 2017 Aug;207:117–24. doi: 10.1016/j.vetmic.2017.06.002 (PMC5548070; doi:10.1016/j.vetmic.2017.06.002)
Supplement: Supplementary file 2 [file mmc2.docx]

## SUPPLEMENTARY TABLES

### Supplementary table S1a. MIC distribution of clinical case (CC) isolates from 2009-2011 (Table S1a), CC isolates from 2013-2014 (Table S1b), non-clinical case isolates (NCC) from 2009-2011 (Table S1c) and NCC from 2013-2014 (Table S1d).

|  | *S. suis* (n=93) | | | | | | | | | | | | | | | | | | | | | | | | |
| --- | --- | --- | --- | --- | --- | --- | --- | --- | --- | --- | --- | --- | --- | --- | --- | --- | --- | --- | --- | --- | --- | --- | --- | --- | --- |
| Antimicrobial agent | MIC values (µg/mL) | | | | | | | | | | | | | | | | | | | | MIC_50_  (µg/mL) | MIC_90_  (µg/mL) | S (%) | I (%) | R (%) |
|  | 0.002 | 0.004 | 0.008 | 0.015 | 0.03 | 0.06 | 0.12 | 0.25 | 0.5 | 1 | 2 | 4 | 8 | 16 | 32 | 64 | 128 | 256 | 512 | 1024 |  |  |  |  |  |
| **Amoxicillin** |  |  |  |  | 90 | 2 | 0 | 0 | 0 | 1 | 0 | 0 | 0 | 0 |  |  |  |  |  |  | ≤0.03 | ≤0.03 |  |  |  |
| **Amoxicillin/ Clavulanate*** |  |  |  |  | 91 | 1 | 0 | 0 | 0 | 1 | 0 | 0 | 0 | 0 | 0 |  |  |  |  |  | ≤0.03 | ≤0.03 | 100% | 0% | 0% |
| **Penicillin** |  |  |  |  | 83 | 0 | 5 | 1 | 1 | 1 | 1 | 0 | 0 | 0 | 0 | 0 |  |  |  |  | ≤0.03 | ≤0.03 | 97% | 1% | 2% |
| **Cefquinome** | 3 | 0 | 18 | 47 | 21 | 2 | 0 | 1 | 1 | 0 | 0 | 0 |  |  |  |  |  |  |  |  | 0.015 | 0.03 |  |  |  |
| **Ceftiofur** |  |  | 0 | 0 | 1 | 14 | 65 | 5 | 6 | 0 | 0 | 1 | 1 | 0 |  |  |  |  |  |  | 0.12 | 0.25 | 98% | 1% | 1% |
| **Doxycycline** |  |  |  |  | 0 | 7 | 17 | 2 | 1 | 0 | 0 | 0 | 36 | 30 | 0 | 0 |  |  |  |  | 8 | 16 |  |  |  |
| **Tetracycline** |  |  |  |  |  | 0 | 0 | 1 | 6 | 14 | 6 | 0 | 0 | 0 | 32 | 33 | 1 |  |  |  | 32 | 64 | 8% | 15% | 77% |
| **Tiamulin** |  |  |  |  | 0 | 1 | 0 | 2 | 3 | 45 | 32 | 0 | 4 | 0 | 2 | 2 | 2 |  |  |  | 1 | 8 |  |  |  |
| **Enrofloxacin** |  | 0 | 0 | 0 | 0 | 0 | 2 | 24 | 66 | 1 | 0 | 0 | 0 |  |  |  |  |  |  |  | 0.5 | 0.5 | 99% | 1% | 0% |
| **Marbofloxacin** |  |  | 0 | 11 | 0 | 0 | 0 | 2 | 24 | 56 | 0 | 0 | 0 | 0 |  |  |  |  |  |  | 1 | 1 |  |  |  |
| **Trimethoprim/ Sulfamethoxazole**** |  |  |  | 4 | 22 | 45 | 6 | 4 | 2 | 1 | 3 | 4 | 1 | 0 | 0 | 1 |  |  |  |  | 0.06 | 1 | 89% | 4% | 6% |
| **Tilmicosin** |  |  |  |  |  | 0 | 0 | 0 | 0 | 0 | 6 | 44 | 0 | 0 | 1 | 0 | 0 | 42 |  |  | 4 | >128 |  |  |  |
| **Tylosin** |  |  |  |  |  |  | 0 | 1 | 35 | 13 | 1 | 1 | 0 | 0 | 0 | 0 | 1 | 0 | 41 |  | 1 | >256 |  |  |  |
| **Erythromycin** |  |  |  | 20 | 29 | 1 | 1 | 0 | 0 | 0 | 2 | 1 | 0 | 1 | 1 | 37 |  |  |  |  | 0.03 | >32 | 55% | 0% | 45% |
| **Lincomycin** |  |  |  |  |  | 1 | 2 | 9 | 7 | 19 | 7 | 1 | 3 | 1 | 0 | 2 | 10 | 31 |  |  | 8 | >128 |  |  |  |
| **Spectinomycin** |  |  |  |  |  |  |  | 0 | 0 | 0 | 0 | 2 | 22 | 59 | 7 | 0 | 0 | 0 | 0 | 3 | 16 | 32 |  |  |  |
| **Florfenicol** |  |  |  |  | 0 | 0 | 0 | 0 | 0 | 14 | 76 | 3 | 0 | 0 | 0 | 0 |  |  |  |  | 2 | 2 | 97% | 3% | 0% |

Note: White cells indicate the dilution range tested. Values in the grey indicate MIC values over the highest concentration in the tested range. Green and red vertical lines respectively describe the sensitive and resistant clinical breakpoints recommended by the CLSI (2013).

* Amoxicillin/Clavulanate combination was tested in a concentration ratio of 2:1. MIC values in the table represent Amoxicillin concentrations.

** Trimethoprim/Sulfamethoxazole combination was tested in a concentration ratio of 1:2. MIC values in the table represent Sulfamethoxazol concentrations.

### Supplementary table S1b. MIC distribution of clinical case (CC) isolates from 2013-2014.

|  | *S. suis* (n=117) | | | | | | | | | | | | | | | | | | | | | | | | |
| --- | --- | --- | --- | --- | --- | --- | --- | --- | --- | --- | --- | --- | --- | --- | --- | --- | --- | --- | --- | --- | --- | --- | --- | --- | --- |
| Antimicrobial agent | MIC values (µg/mL) | | | | | | | | | | | | | | | | | | | | MIC_50_  (µg/mL) | MIC_90_  (µg/mL) | S (%) | I (%) | R (%) |
|  | 0.002 | 0.004 | 0.008 | 0.015 | 0.03 | 0.06 | 0.12 | 0.25 | 0.5 | 1 | 2 | 4 | 8 | 16 | 32 | 64 | 128 | 256 | 512 | 1024 |  |  |  |  |  |
| **Amoxicillin** |  |  |  |  | 115 | 2 | 0 | 0 | 0 | 0 | 0 | 0 | 0 | 0 |  |  |  |  |  |  | ≤0.03 | ≤0.03 |  |  |  |
| **Amoxicillin/ Clavulanate*** |  |  |  |  | 115 | 2 | 0 | 0 | 0 | 0 | 0 | 0 | 0 | 0 | 0 |  |  |  |  |  | ≤0.03 | ≤0.03 | 100% | 0% | 0% |
| **Penicillin** |  |  |  |  | 100 | 1 | 5 | 8 | 2 | 1 | 0 | 0 | 0 | 0 | 0 | 0 |  |  |  |  | ≤0.03 | 0.12 | 97% | 2% | 1% |
| **Cefquinome** | 0 | 0 | 14 | 51 | 42 | 10 | 0 | 0 | 0 | 0 | 0 | 0 |  |  |  |  |  |  |  |  | 0.015 | 0.03 |  |  |  |
| **Ceftiofur** |  |  | 0 | 0 | 0 | 16 | 67 | 17 | 14 | 3 | 0 | 0 | 0 | 0 |  |  |  |  |  |  | 0.12 | 0.5 | 100% | 0% | 0% |
| **Doxycycline** |  |  |  |  | 0 | 6 | 14 | 1 | 1 | 0 | 0 | 1 | 24 | 60 | 9 | 1 |  |  |  |  | 16 | 16 |  |  |  |
| **Tetracycline** |  |  |  |  |  | 0 | 0 | 1 | 3 | 10 | 8 | 0 | 0 | 0 | 18 | 69 | 8 |  |  |  | 64 | 64 | 3% | 9% | 88% |
| **Tiamulin** |  |  |  |  | 0 | 0 | 0 | 0 | 6 | 51 | 33 | 4 | 2 | 9 | 4 | 6 | 2 |  |  |  | 2 | 32 |  |  |  |
| **Enrofloxacin** |  | 0 | 0 | 0 | 0 | 0 | 1 | 38 | 74 | 3 | 0 | 1 | 0 |  |  |  |  |  |  |  | 0.5 | 0.5 | 97% | 3% | 1% |
| **Marbofloxacin** |  |  | 0 | 0 | 0 | 0 | 0 | 1 | 38 | 70 | 7 | 0 | 0 | 1 |  |  |  |  |  |  | 1 | 1 |  |  |  |
| **Trimethoprim/ Sulfamethoxazole**** |  |  |  | 3 | 19 | 41 | 2 | 12 | 13 | 4 | 5 | 2 | 5 | 6 | 2 | 3 |  |  |  |  | 0.06 | 8 | 77% | 8% | 15% |
| **Tilmicosin** |  |  |  |  |  | 0 | 0 | 0 | 1 | 0 | 6 | 45 | 0 | 0 | 0 | 1 | 0 | 64 |  |  | 256 | >128 |  |  |  |
| **Tylosin** |  |  |  |  |  |  | 0 | 1 | 32 | 19 | 0 | 1 | 0 | 0 | 0 | 0 | 0 | 1 | 63 |  | >256 | >256 |  |  |  |
| **Erythromycin** |  |  |  | 7 | 39 | 7 | 0 | 0 | 0 | 0 | 0 | 1 | 2 | 0 | 0 | 61 |  |  |  |  | >32 | >32 | 45% | 0% | 55% |
| **Lincomycin** |  |  |  |  |  | 0 | 0 | 9 | 10 | 12 | 15 | 1 | 1 | 1 | 2 | 0 | 16 | 50 |  |  | 128 | >128 |  |  |  |
| **Spectinomycin** |  |  |  |  |  |  |  | 0 | 0 | 0 | 0 | 3 | 16 | 76 | 6 | 0 | 0 | 0 | 0 | 16 | 16 | >512 |  |  |  |
| **Florfenicol** |  |  |  |  | 0 | 0 | 0 | 0 | 1 | 18 | 91 | 7 | 0 | 0 | 0 | 0 |  |  |  |  | 2 | 2 | 94% | 6% | 0% |

Note: White cells indicate the dilution range tested. Values in the grey indicate MIC values over the highest concentration in the tested range. Green and red vertical lines respectively describe the sensitive and resistant clinical breakpoints recommended by the CLSI (2013).

* Amoxicillin/Clavulanate combination was tested in a concentration ratio of 2:1. MIC values in the table represent Amoxicillin concentrations.

** Trimethoprim/Sulfamethoxazole combination was tested in a concentration ratio of 1:2. MIC values in the table represent Sulfamethoxazol concentrations.

|  | *S. suis* (n=66) | | | | | | | | | | | | | | | | | | | | | | | | |
| --- | --- | --- | --- | --- | --- | --- | --- | --- | --- | --- | --- | --- | --- | --- | --- | --- | --- | --- | --- | --- | --- | --- | --- | --- | --- |
| Antimicrobial agent | MIC values (µg/mL) | | | | | | | | | | | | | | | | | | | | MIC_50_  (µg/mL) | MIC_90_  (µg/mL) | S (%) | I (%) | R (%) |
|  | 0.002 | 0.004 | 0.008 | 0.015 | 0.03 | 0.06 | 0.12 | 0.25 | 0.5 | 1 | 2 | 4 | 8 | 16 | 32 | 64 | 128 | 256 | 512 | 1024 |  |  |  |  |  |
| **Amoxicillin** |  |  |  |  | 61 | 3 | 0 | 0 | 0 | 0 | 1 | 1 | 0 | 0 |  |  |  |  |  |  | ≤0.03 | ≤0.03 |  |  |  |
| **Amoxicillin/ Clavulanate*** |  |  |  |  | 61 | 3 | 0 | 0 | 0 | 0 | 1 | 1 | 0 | 0 | 0 |  |  |  |  |  | ≤0.03 | ≤0.03 | 100% | 0% | 0% |
| **Penicillin** |  |  |  |  | 43 | 4 | 7 | 4 | 2 | 3 | 2 | 1 | 0 | 0 | 0 | 0 |  |  |  |  | ≤0.03 | 0.5 | 88% | 3% | 9% |
| **Cefquinome** | 2 | 0 | 12 | 20 | 14 | 11 | 4 | 2 | 1 | 0 | 0 | 0 |  |  |  |  |  |  |  |  | 0.015 | 0.12 |  |  |  |
| **Ceftiofur** |  |  | 0 | 0 | 0 | 11 | 25 | 8 | 7 | 9 | 3 | 2 | 1 | 0 |  |  |  |  |  |  | 0.12 | 1 | 95% | 3% | 2% |
| **Doxycycline** |  |  |  |  | 0 | 1 | 10 | 1 | 0 | 0 | 0 | 0 | 14 | 39 | 1 | 0 |  |  |  |  | 16 | 16 |  |  |  |
| **Tetracycline** |  |  |  |  |  | 0 | 0 | 0 | 0 | 2 | 8 | 2 | 0 | 0 | 5 | 40 | 8 | 1 |  |  | 64 | 128 | 0% | 3% | 97% |
| **Tiamulin** |  |  |  |  | 0 | 0 | 2 | 6 | 2 | 13 | 13 | 8 | 4 | 4 | 9 | 3 | 2 |  |  |  | 2 | 32 |  |  |  |
| **Enrofloxacin** |  | 0 | 0 | 0 | 0 | 0 | 0 | 26 | 37 | 1 | 1 | 0 | 0 | 1 |  |  |  |  |  |  | 0.5 | 0.5 | 95% | 2% | 3% |
| **Marbofloxacin** |  |  | 0 | 9 | 0 | 0 | 0 | 0 | 27 | 30 | 0 | 0 | 0 | 0 |  |  |  |  |  |  | 0.5 | 1 |  |  |  |
| **Trimethoprim/ Sulfamethoxazole**** |  |  |  | 6 | 15 | 6 | 7 | 8 | 11 | 2 | 2 | 6 | 0 | 0 | 0 | 3 |  |  |  |  | 0.12 | 4 | 80% | 6% | 14% |
| **Tilmicosin** |  |  |  |  |  | 0 | 0 | 0 | 0 | 0 | 6 | 33 | 3 | 0 | 0 | 0 | 0 | 24 |  |  | 4 | >128 |  |  |  |
| **Tylosin** |  |  |  |  |  |  | 0 | 0 | 22 | 19 | 1 | 0 | 0 | 0 | 0 | 0 | 0 | 0 | 24 |  | 1 | >256 |  |  |  |
| **Erythromycin** |  |  |  | 10 | 23 | 7 | 0 | 0 | 0 | 0 | 0 | 1 | 2 | 0 | 1 | 22 |  |  |  |  | 0.06 | >32 | 61% | 0% | 39% |
| **Lincomycin** |  |  |  |  |  | 1 | 5 | 1 | 6 | 11 | 5 | 3 | 8 | 1 | 0 | 0 | 2 | 23 |  |  | 8 | >128 |  |  |  |
| **Spectinomycin** |  |  |  |  |  |  |  | 0 | 0 | 0 | 0 | 1 | 15 | 34 | 14 | 0 | 0 | 0 | 1 | 1 | 16 | 32 |  |  |  |
| **Florfenicol** |  |  |  |  | 0 | 0 | 0 | 0 | 0 | 14 | 51 | 1 | 0 | 0 | 0 | 0 |  |  |  |  | 2 | 2 | 98% | 2% | 0% |

### Supplementary table S1c. MIC distribution of non-clinical case (NCC) isolates from 2009-2011.

Note: White cells indicate the dilution range tested. Values in the grey indicate MIC values over the highest concentration in the tested range. Green and red vertical lines respectively describe the sensitive and resistant clinical breakpoints recommended by the CLSI (2013).

* Amoxicillin/Clavulanate combination was tested in a concentration ratio of 2:1. MIC values in the table represent Amoxicillin concentrations.

** Trimethoprim/Sulfamethoxazole combination was tested in a concentration ratio of 1:2. MIC values in the table represent Sulfamethoxazol concentrations.

|  | *S. suis* (n=129) | | | | | | | | | | | | | | | | | | | | | | | | |
| --- | --- | --- | --- | --- | --- | --- | --- | --- | --- | --- | --- | --- | --- | --- | --- | --- | --- | --- | --- | --- | --- | --- | --- | --- | --- |
| Antimicrobial agent | MIC values (µg/mL) | | | | | | | | | | | | | | | | | | | | MIC_50_  (µg/mL) | MIC_90_  (µg/mL) | S (%) | I (%) | R (%) |
|  | 0.002 | 0.004 | 0.008 | 0.015 | 0.03 | 0.06 | 0.12 | 0.25 | 0.5 | 1 | 2 | 4 | 8 | 16 | 32 | 64 | 128 | 256 | 512 | 1024 |  |  |  |  |  |
| **Amoxicillin** |  |  |  |  | 116 | 7 | 3 | 0 | 3 | 0 | 0 | 0 | 0 | 0 |  |  |  |  |  |  | ≤0.03 | 0.06 |  |  |  |
| **Amoxicillin/ Clavulanate*** |  |  |  |  | 114 | 8 | 3 | 1 | 3 | 0 | 0 | 0 | 0 | 0 | 0 |  |  |  |  |  | ≤0.03 | 0.06 | 100% | 0% | 0% |
| **Penicillin** |  |  |  |  | 80 | 8 | 7 | 12 | 12 | 7 | 3 | 0 | 0 | 0 | 0 | 0 |  |  |  |  | ≤0.03 | 0.5 | 83% | 9% | 8% |
| **Cefquinome** | 0 | 2 | 7 | 50 | 37 | 26 | 4 | 2 | 1 | 0 | 0 | 0 |  |  |  |  |  |  |  |  | 0.03 | 0.06 |  |  |  |
| **Ceftiofur** |  |  | 0 | 0 | 1 | 7 | 58 | 17 | 23 | 17 | 4 | 2 | 0 | 0 |  |  |  |  |  |  | 0.12 | 1 | 98% | 2% | 0% |
| **Doxycycline** |  |  |  |  | 0 | 1 | 18 | 1 | 0 | 0 | 0 | 7 | 48 | 52 | 2 | 0 |  |  |  |  | 8 | 16 |  |  |  |
| **Tetracycline** |  |  |  |  |  | 0 | 0 | 0 | 0 | 0 | 16 | 4 | 0 | 3 | 31 | 60 | 9 | 6 |  |  | 64 | 128 | 0% | 0% | 100% |
| **Tiamulin** |  |  |  |  | 0 | 0 | 5 | 23 | 5 | 15 | 24 | 6 | 18 | 14 | 13 | 5 | 1 |  |  |  | 2 | 32 |  |  |  |
| **Enrofloxacin** |  | 0 | 0 | 0 | 0 | 0 | 7 | 54 | 62 | 6 | 0 | 0 | 0 |  |  |  |  |  |  |  | 0.5 | 0.5 | 95% | 5% | 0% |
| **Marbofloxacin** |  |  | 0 | 0 | 0 | 0 | 0 | 3 | 62 | 60 | 4 | 0 | 0 | 0 |  |  |  |  |  |  | 0.5 | 1 |  |  |  |
| **Trimethoprim/ Sulfamethoxazole**** |  |  |  | 13 | 27 | 5 | 8 | 23 | 17 | 11 | 11 | 6 | 3 | 4 | 0 | 1 |  |  |  |  | 0.25 | 4 | 72% | 17% | 11% |
| **Tilmicosin** |  |  |  |  |  | 0 | 0 | 0 | 0 | 1 | 17 | 54 | 3 | 1 | 0 | 0 | 0 | 53 |  |  | 4 | >128 |  |  |  |
| **Tylosin** |  |  |  |  |  |  | 0 | 0 | 53 | 22 | 0 | 1 | 0 | 0 | 0 | 0 | 1 | 1 | 51 |  | 1 | >256 |  |  |  |
| **Erythromycin** |  |  |  | 10 | 58 | 6 | 1 | 0 | 0 | 0 | 2 | 5 | 3 | 0 | 1 | 43 |  |  |  |  | 0.03 | >32 | 58% | 0% | 42% |
| **Lincomycin** |  |  |  |  |  | 2 | 22 | 0 | 1 | 21 | 5 | 7 | 13 | 2 | 0 | 0 | 3 | 53 |  |  | 8 | >128 |  |  |  |
| **Spectinomycin** |  |  |  |  |  |  |  | 0 | 0 | 0 | 0 | 2 | 31 | 66 | 26 | 1 | 0 | 0 | 0 | 3 | 16 | 32 |  |  |  |
| **Florfenicol** |  |  |  |  | 0 | 0 | 0 | 0 | 0 | 36 | 93 | 0 | 0 | 0 | 0 | 0 |  |  |  |  | 2 | 2 | 100% | 0% | 0% |

### Supplementary table S1d. MIC distribution of non-clinical case (NCC) isolates from 2013-2014.

Note: White cells indicate the dilution range tested. Values in the grey indicate MIC values over the highest concentration in the tested range. Green and red vertical lines respectively describe the sensitive and resistant clinical breakpoints recommended by the CLSI (2013).

* Amoxicillin/Clavulanate combination was tested in a concentration ratio of 2:1. MIC values in the table represent Amoxicillin concentrations.

** Trimethoprim/Sulfamethoxazole combination was tested in a concentration ratio of 1:2. MIC values in the table represent Sulfamethoxazol concentrations.

### Supplementary table S2. Profile of multiple non wild-type combinations for clinical and non-clinical isolates in 2009-2011 and 2013-2014.

| Multiple antimicrobial class NWT profile | Number  of NWT | Full collection (n=405) | 2009-2011 | |  | 2013-2014 | |
| --- | --- | --- | --- | --- | --- | --- | --- |
|  |  |  | CC n= 93 | NCC n= 66 |  | CC n= 117 | NCC n= 129 |
| MAC TET FQ LIN PEN SPE TIA TMPS | 8 | 3.0% | 2.2% | 1.5% |  | 7.7% | 0.0% |
| MAC TET FQ LIN PEN TIA TMPS | 7 | 9.4% | 2.2% | 12.1% |  | 3.4% | 18.6% |
| MAC TET FQ LIN SPE TIA TMPS | 7 | 1.7% | 0.0% | 0.0% |  | 4.3% | 1.6% |
| TET FQ LIN PEN SPE TIA TMPS | 7 | 0.2% | 0.0% | 1.5% |  | 0.0% | 0.0% |
| MAC TET FQ LIN PEN TMPS | 6 | 2.0% | 0.0% | 4.5% |  | 1.7% | 2.3% |
| MAC TET FQ LIN PEN TIA | 6 | 0.7% | 0.0% | 1.5% |  | 0.9% | 0.8% |
| MAC TET FQ LIN TIA TMPS | 6 | 1.5% | 1.1% | 3.0% |  | 0.9% | 1.6% |
| TET FQ LIN PEN TIA TMPS | 6 | 3.2% | 1.1% | 6.1% |  | 0.0% | 6.2% |
| TET FQ LIN SPE TIA TMPS | 6 | 0.7% | 0.0% | 0.0% |  | 1.7% | 0.8% |
| FQ LIN PEN TIA TMPS | 5 | 1.0% | 1.1% | 1.5% |  | 0.0% | 1.6% |
| MAC FQ LIN TIA TMPS | 5 | 0.5% | 0.0% | 0.0% |  | 0.0% | 1.6% |
| MAC TET FQ LIN PEN | 5 | 1.2% | 2.2% | 1.5% |  | 0.0% | 1.6% |
| MAC TET FQ LIN TMPS | 5 | 4.4% | 4.3% | 3.0% |  | 8.5% | 1.6% |
| MAC TET FQ LIN TIA | 5 | 1.2% | 1.1% | 3.0% |  | 0.0% | 1.6% |
| TET FQ LIN PEN TMPS | 5 | 1.5% | 1.1% | 0.0% |  | 0.9% | 3.1% |
| TET FQ LIN PEN TIA | 5 | 1.0% | 0.0% | 1.5% |  | 0.0% | 2.3% |
| FQ LIN PEN TMPS | 4 | 0.2% | 0.0% | 1.5% |  | 0.0% | 0.0% |
| FQ LIN PEN TIA | 4 | 0.2% | 0.0% | 0.0% |  | 0.0% | 0.8% |
| FQ LIN SPE TIA | 4 | 0.2% | 1.1% | 0.0% |  | 0.0% | 0.0% |
| FQ LIN TIA TMPS | 4 | 0.7% | 0.0% | 0.0% |  | 1.7% | 0.8% |
| MAC FQ LIN TMPS | 4 | 0.7% | 0.0% | 0.0% |  | 1.7% | 0.8% |
| MAC FQ LIN TIA | 4 | 0.2% | 0.0% | 1.5% |  | 0.0% | 0.0% |
| MAC TET FQ LIN | 4 | 19.0% | 32.3% | 7.6% |  | 24.8% | 10.1% |
| TET FQ LIN PEN | 4 | 0.5% | 0.0% | 1.5% |  | 0.0% | 0.8% |
| TET FQ LIN TMPS | 4 | 7.7% | 2.2% | 6.1% |  | 6.0% | 14.0% |
| TET FQ LIN TIA | 4 | 1.0% | 0.0% | 3.0% |  | 0.0% | 1.6% |
| TET FQ LIN TIA TMPS | 4 | 2.5% | 1.1% | 4.5% |  | 1.7% | 3.1% |
| FQ LIN TMPS | 3 | 1.7% | 1.1% | 1.5% |  | 3.4% | 0.8% |
| FQ LIN TIA | 3 | 1.5% | 0.0% | 4.5% |  | 0.9% | 1.6% |
| MAC FQ LIN | 3 | 0.5% | 0.0% | 0.0% |  | 1.7% | 0.0% |
| TET FQ LIN | 3 | 8.4% | 9.7% | 13.6% |  | 12.0% | 1.6% |
| TET FQ TMPS | 3 | 0.5% | 0.0% | 1.5% |  | 0.9% | 0.0% |
| FQ LIN | 2 | 8.6% | 23.7% | 3.0% |  | 8.5% | 0.8% |
| FQ TMPS | 2 | 0.2% | 0.0% | 0.0% |  | 0.0% | 0.8% |
| TET FQ | 2 | 8.6% | 10.8% | 4.5% |  | 6.0% | 11.6% |
| FQ | 1 | 3.5% | 2.2% | 4.5% |  | 0.9% | 6.2% |

Note: MAC for macrolides, TET for tetracyclines, FQ for fluoroquinolones, LIN for lincosamides, PEN for penams, SPE for (spectinomycin) aminoglycosides, TIA for (tiamulin) pleuromutilins and TMPS for trimethoprim/sulfamethoxazole. Underlined those profiles including MAC, TET, FQ and LIN.
